# Efficient glyceric acid electrosynthesis from waste glycerol on rare-earth-metal-alloyed mesoporous PtPb nanosheets

**DOI:** 10.1093/nsr/nwaf343

**Published:** 2025-08-19

**Authors:** Dongping Fan, Lizhi Sun, Ruijia Yuan, Zhen-An Qiao, Shunai Che, Ben Liu

**Affiliations:** Key Laboratory of Green Chemistry and Technology of Ministry of Education, National and Local Joint Engineering Laboratory of Energy Plant Biofuel Preparation and Utilization, College of Chemistry, Sichuan University, Chengdu 610064, China; Key Laboratory of Green Chemistry and Technology of Ministry of Education, National and Local Joint Engineering Laboratory of Energy Plant Biofuel Preparation and Utilization, College of Chemistry, Sichuan University, Chengdu 610064, China; Key Laboratory of Green Chemistry and Technology of Ministry of Education, National and Local Joint Engineering Laboratory of Energy Plant Biofuel Preparation and Utilization, College of Chemistry, Sichuan University, Chengdu 610064, China; State Key Laboratory of Inorganic Synthesis and Preparative Chemistry, Jilin University, Changchun 130012, China; School of Chemistry and Chemical Engineering, Frontiers Science Center for Transformative Molecules, State Key Laboratory of Composite Materials, Shanghai Key Laboratory for Molecular Engineering of Chiral Drugs, Shanghai Jiao Tong University, Shanghai 200240, China; Key Laboratory of Green Chemistry and Technology of Ministry of Education, National and Local Joint Engineering Laboratory of Energy Plant Biofuel Preparation and Utilization, College of Chemistry, Sichuan University, Chengdu 610064, China

**Keywords:** mesoporous materials, rare-earth metals, electrocatalysis, glyceric acid, glycerol

## Abstract

The electrocatalytic glycerol oxidation reaction (GOR) offers a promising route to synthesize high value-added chemicals, for example glyceric acid (GLA), in an economic and sustainable manner. Despite some great achievements, GLA selectivity and yield rate of GOR electrocatalysis remain unsatisfactory due to uncontrollable C–C bond cleavage of glycerol (for unfavorable C1 and C2 products). In this work, rare-earth-metal (REM)-alloyed PtPb mesoporous nanosheets are demonstrated as novel yet high-performance electrocatalysts for selective GLA electrosynthesis from GOR. The best electrocatalyst—PtPbY MNSs—delivers outstanding performance for GLA electrosynthesis from glycerol, including superior selectivity of 72.5% and recordable yield rate of 656 μmol mg_cat_^−1^ h^−1^, surpassing most electrocatalysts reported in the literature. Meanwhile, PtPbY MNSs hold impressive stability of GOR electrocatalysis, retaining the high GLA selectivity and yield rate for reaching 15 cycles. Superior performance, revealed by *in situ* characterizations and density functional theory calculations, is ascribed to structural and compositional synergies that kinetically promote the reactivity of glycerol and accelerate the desorption of GLA while minimizing undesirable C–C bond cleavage. This work elaborates a powerful alternative for selective electrosynthesis of high-value-added chemicals from waste alcohols by optimizing chemisorption properties of mesoporous metals by REM alloys.

## Teaser

This work elaborates a powerful strategy for selective electrosynthesis of high value-added glyceric acid from waste glycerol by optimizing chemisorption properties of mesoporous metals with REM alloys.

## INTRODUCTION

**Figure 1.**
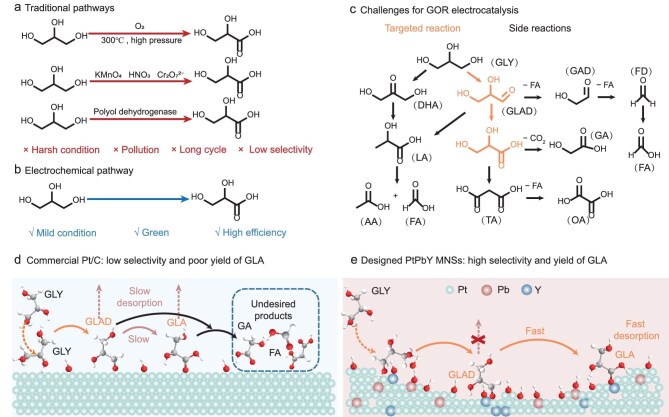
Schematic illustration of electrocatalytic glycerol-to-GLA conversion. (a) Traditional pathways and (b) electrochemical pathway for GLA synthesis from waste glycerol. (c) Challenges of GOR electrocatalysis with different products. (d) Commercial Pt electrocatalysts for various products of GOR electrocatalysis. (e) Designed REM-alloyed electrocatalysts for selective GLA electrosynthesis of GOR electrocatalysis.

Glyceric acid (GLA), as a highly functionalized organic acid, is an important chemical intermediate that participates in various synthetic reactions such as esterification, polymerization, and dehydration, holding significant value in the fields of chemistry, biology, and food [1,2]. The global market for GLA was estimated to be worth $12 million in 2024 and is projected to be of an adjusted size of $19.2 million by 2031 [3]. Currently, thermal catalysis is utilized to synthesize GLA by the selective glycerol oxidation reaction (GOR) [4–7]. Despite industrial importance, it requires harsh reaction conditions (high temperature and pressure, strong oxidants, and undesired pollutants) and holds low selectivity and cycling (Fig. 1a). In comparison, GOR electrocatalysis with water as the oxidant offers a green and sustainable route to efficiently synthesize GLA under ambient conditions, which potentially addresses the shortcomings of traditional pathways (Fig. 1b). However, glycerol (GLY) chemically composed of polyhydroxy groups can be electrochemically oxidized into various C3 products, including GLA [8–10], glyceraldehyde (GLAD) [11,12], dihydroxyacetone (DHA) [13–15], lactic acid (LA) [16–18], and tartronic acid (TA) [19]. Meanwhile, other side reactions of GOR electrocatalysis involved undesired C–C bond cleavage also form some C1 and C2 byproducts (glycolic acid (GA) [20,21], oxalic acid (OA) [22,23], acetic acid (AA), and formic acid (FA [24–28])) (Fig. 1c) [29–31]. They thus result in low selectivity and poor GLA yield rate from GOR electrocatalysis, especially when considering its high potential in industrial production.

In selective GLA electrosynthesis, GOR involves the dehydrogenation, by OH^−^, of primary hydroxyl groups (C–OH) and C–H glycerol bonds to form GLAD as an intermediate. Subsequently, activation of the C=O bond in GLAD is followed by the insertion of O–H and further dehydrogenation to form GLA [32–34]. Over the past few years, different kinds of metal electrocatalysts, including precious metals (Pt [18,35,36], Pd [32,37], and Au [30,38]) and non-precious metal (Ni [14,39]), have been employed for selective GLA electrosynthesis from GOR. Because of the lower *d*-band center, in comparison, Pt is considered superior for GOR electrocatalysis [40,41]. Nevertheless, inappropriate adsorption of GLAD and strong adsorption of GLA on Pt sites hinder the oxidative conversion of GLAD to GLA and the desorption of GLA, which, more importantly, provide more opportunities for deeper oxidation cleavage of the C–C bond into undesired C1/C2 products (Fig. 1d) [42]. Encouragingly, some strategies, including alloying with other 3*d* metals and coordinating with organic/inorganic groups, have been proposed to optimize chemisorption properties of Pt sites and further promote GLA electrosynthesis [43,44]. In spite of some encouraging progress, the improvements in both GLA selectivity and yield rate remain unsatisfactory. Therefore, it is necessary to develop novel high-performance electrocatalysts to optimize the chemisorption properties and further regulate the reaction pathways for selective GLA electrosynthesis from GOR while minimizing undesirable C–C bond cleavage.

In this work, we propose rare-earth-metal (REM) alloys to optimize chemisorption properties of two-dimensional (2D) PtPb mesoporous nanosheets (MNSs) for selective GLA electrosynthesis from GOR in an alkaline medium (Fig. 1e). On the one hand, REMs are deeply embedded in the atomic core of Pt and allow chemical change interactions with reactive species [45–47]. On the other hand, a mesoporous microenvironment with concave metal sites imitates enzymatic structural functions and further regulates the chemisorption behaviors of reactant OH^−^ for selective dehydrogenation of GLY intermediate [48–51]. After experimentally evaluating 14 types of REMs, PtPbY MNSs enable the best electrocatalytic performance for selective GLA electrosynthesis from GOR, which is remarkably better than other reported electrocatalysts. Mechanism studies combined with *in situ* spectroscopic techniques and density functional theory (DFT) calculations reveal that the synergies of Y alloys and mesoporous structures kinetically promote the dehydrogenation of GLY for GLA electrosynthesis while inhibiting further oxidation cleavage of C–C bonds.

## RESULTS AND DISCUSSION

**Figure 2.**
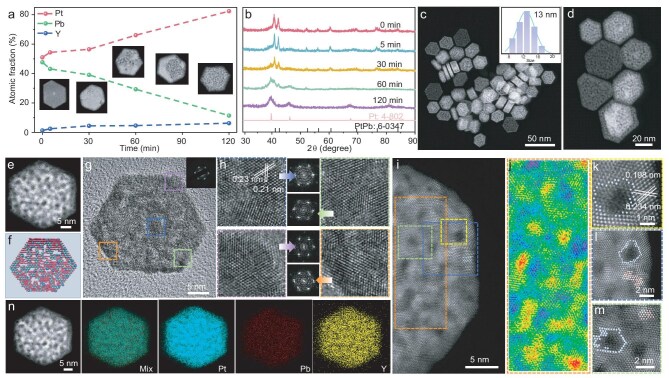
Physicochemical characterizations. (a) Atomic fractions of metals and (b) powder XRD patterns of PtPbY MNSs synthesized with different etching times. (c) Low-magnification and (d) high-magnification HAADF-STEM images, (e) HAADF-STEM image and (f) simulated schematic image, (g) high-resolution TEM and corresponding SAED/FFT patterns, (h) high-resolution TEM images, (i) high-resolution HAADF-STEM image, (j) equalized rainbow color mode image, (k–m) enlarged HDDAF-STEM images, and (n) HAADF-STEM EDX mapping images of 2D PtPbY MNSs.

Two-dimensional REM-alloyed PtPb MNSs were prepared by a facile two-step selective etching strategy. First, REM-alloyed PtPb nanosheets with a solid surface and L1_0_ intermetallic phase were first obtained and utilized as the parent template by a solvothermal method with a modified route as reported in the literature [52]. Then, concentrated HNO_3_ was injected into the above solution to enable selective etching of active metals and further form abundant mesopores in the absence of any mesopore-forming template, based on the different dealloying rates of the metal sources. With Y-alloyed PtPb (PtPbY) MNSs as an example (the best performance as discussed below), after being reacted for 120 min, Pb atoms were etched out, followed by a quick decrease in the atomic fraction from 47.7% to 11.1% (Fig. 2a). In comparison, both Pt and Y fractions were correspondingly increased during the etching process. The atomic ratio of Pt:Pb:Y in the final PtPbY MNSs after 2 h of etching was 82.4:11.4:6.2, which was similar to the result collected from the inductively coupled plasma mass spectrometry (85.2:9.4:5.4) (Fig. S1). Meanwhile, some uneven pores gradually appeared on the surface of solid nanosheets and further formed penetrated mesopores for 120 min, as shown by the high angle annular dark-field scanning transmission electron microscopy (HAADF-STEM) images (insets in Fig. 2a and Fig. S2). Meanwhile, powder X-ray diffraction (XRD) patterns disclosed that the intermetallic L1_0_ phase gradually evolved into mixed phases and finally to random alloys during the etching process (Fig. 2b). Finally, PtPbY MNSs with surface-clean metal sites were collected by being washed several times with ethanol/cyclohexane (see the Experimental Section for details). *In situ* structural characterizations and more controlled experiments further confirmed that metal mesopores were formed by selective etching of active Pb atoms while retaining a quasi-crystalline structure (Figs S3–S5). Similarly, 2D PtPb MNSs (without REM alloys) and other 13 REM-alloyed PtPb MNSs, including PtPbLa, PtPbCe, PtPbPr, PtPbNd, PtPbSm, PtPbEu, PtPbGd, PtPbTb PtPbDy, PtPbEr, PtPbTm, PtPbYb, and PtPbLu, were prepared using similar procedures but with different intermetallic nanosheets as parent templates (Figs S6–S19).

The physicochemical properties of 2D PtPbY MNSs were then obtained by using some advanced characterization techniques. Low-magnification TEM and HAADF-STEM images showed that the products were nearly hexagonal structures with a high purity and uniform size. The edge length and thickness were estimated to be in the range 7–17 nm and 5–10 nm from >200 samples (Fig. 2c and Fig. S20). High-magnification HAADF-STEM images further displayed high-density and uniform mesopores penetrating the overall nanosheets with mesopore sizes in the range of 2–4 nm (Fig. 2d–f). High-resolution TEM images and corresponding selected-area electron diffraction (SAED) patterns further revealed the atomic structure of a single PtPbY MNS. Interestingly, a nearly single series of spots were observed from the SAED pattern, indicating that they were structurally quasi-single-crystalline (Fig. 2g). Meanwhile, by imaging four randomly selected domains of a MNS, there were almost similar lattice fringes and directions with the same d-spacing distances of 0.23 and 0.21 nm, which were consistent with the (111) and (200) planes of the face-centered cubic (*fcc*) phase (Fig. 2h). In addition to the corresponding fast Fourier transform (FFT) patterns, it can be deduced that 2D PtPbY MNSs were quasi-single-crystalline with a (110) exposed crystalline facet. Aberration corrected high-resolution HAADF-STEM imaging further disclosed the atomic arrangement and mesoporous structure (Fig. 2i). HAADF-STEM rainbow reflection images clearly highlighted the difference in thickness, as represented by different colors, further confirming the existence of high-density mesopores (Fig. 2j). Moreover, atomic orientations observed on the surface of MNS showed a (110) exposed facet. Magnified HAADF-STEM images further confirmed a large number of atomic defects and uncoordinated sites around mesopores (Fig. 2k–m). HAADF-STEM energy dispersive X-ray (EDX) elemental mapping images revealed that all three metals, including Pt, Pb, and Y, were uniformly dispersed in MNS, with a Pt:Pb:Y ratio of 82.4:11.4:6.2, further indicating compositionally alloyed metals (Fig. 2n and Fig. S21). Besides, the Y ratios in PtPbY MNSs were rationally controlled by changing its ratios in the parent PtPbY nanosheets (Fig. S22). The results definitely highlighted successful synthesis of REM-alloyed PtPb MNSs with abundant mesopores, highly active metal sites, quasi-single-crystalline structures, and tunable REMs.

**Figure 3.**
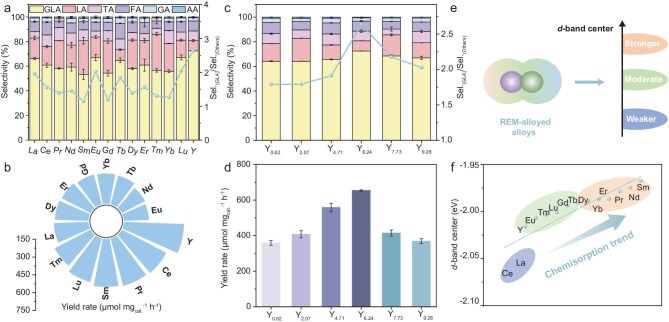
Electrocatalysts screening. (a) Selectivity and (b) GLA yield rates of REM-alloyed PtPb MNSs with different REMs for GOR electrocatalysis. (c) Selectivity and (d) GLA yield rates of PtPbY MNSs with different Y amounts for GOR electrocatalysis. (e) DFT models and (f) *d*-band centers of REM alloys for GOR electrocatalysis.

The electrocatalytic performance of 14 REM-alloyed PtPb MNSs in 1.0 M KOH containing 0.50 M glycerol was first required to evaluate the effect of REMs for GOR. Linear sweep voltammetry (LSV) curves and summarized overpotentials at a current density of 100 mA cm^−2^ showed the huge variations of REM alloys for GOR electrocatalysis (Fig. S23). The products of GOR electrocatalysis were then analyzed by chronoamperometric (CA) measurements using proton nuclear magnetic spectroscopy (^1^H NMR), which were quantified by the external standard calibration curves (Fig. S24). Interestingly, GLA was the predominant product for all types of electrocatalysts, with a high selectivity of >50%. Other byproducts obtained in this work included LA, TA, FA, GA, and AA. Notably, PtPbY MNSs demonstrated the highest GLA selectivity reaching 72.5%, which was 2.64-fold higher than that of all other byproducts (sel._(GLA)_/sel._(others)_) (Fig. 3a). Correspondingly, PtPbY MNSs hold the highest yield rates of GLA, holding a superior value of 656 μmol mg_cat_^−1^ h^−1^ (Fig. 3b). Meanwhile, PtPbY MNSs with different Y ratios were subjected to GOR electrocatalysis (Fig. S25). PtPbY MNSs with a Y ratio of 6.2% exhibited the best GOR performance for GLA electrosynthesis, while increasing or decreasing the Y amounts resulted in simultaneous declines in both electrocatalytic activity and selectivity (Fig. 3c, d).

DFT calculations were then employed to explore the effect of REM alloys on the chemisorption properties during electrocatalysis (Fig. S26). In general, excessively strong chemisorption disfavored product desorption, whereas weaker chemisorption hindered the activity of reactants, both of which potentially decreased electrocatalytic selectivity and activity [53–55]. In comparison, moderate chemisorption favored the adsorption of reactants and desorption of products, which thus enabled the better performance of electrocatalysis (Fig. 3e). According to the *d*-band center theory, six REMs (Y, Eu, Tm, Lu, Gd, and Tb) alloyed with PtPb MNSs disclosed moderate chemisorption properties, which strongly corresponded to high GLA selectivity and activity for GOR electrocatalysis as reported above (Fig. 3f). In comparison to Y, however, the other five REMs possessed deeper oxidation ability for carbon–carbon bond cleavage, which produced more C1/C2 products and thus slightly decreased the selectivity of GLA. Differently, the stronger and weaker *d*-band centers (with other REMs) changed the chemisorption interactions with glycerol and its intermediates (such as GLAD), which also resulted in the weaker or deeper oxidation of glycerol for other products. All results clearly highlighted the high importance of Y alloys, in comparison to other REMs, for selective GLA electrosynthesis from glycerol.

**Figure 4.**
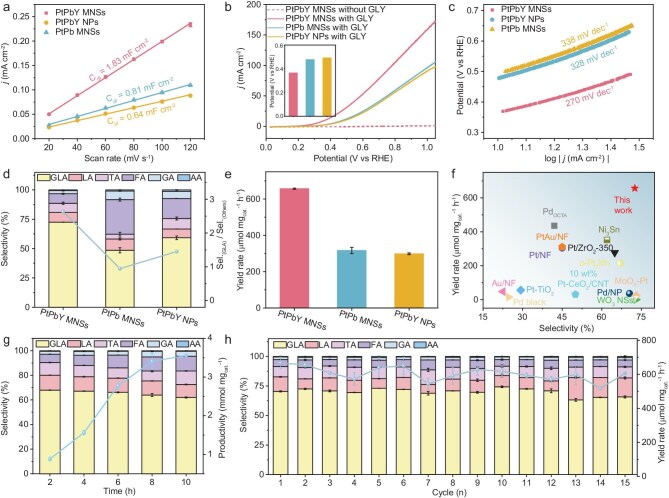
Electrocatalytic performance. (a) Double layer capacitances (C_dl_) values of PtPbY MNSs, PtPb MNSs, and PtPbY NPs collected in 1.0 M KOH. (b) LSV curves and summarized over-potentials, and (c) Tafel plots of PtPbY MNSs, PtPb MNSs, and PtPbY NPs collected in 1.0 M KOH and 0.50 M glycerol. (d) GLA selectivity and (e) yield rates of PtPbY MNSs, PtPb MNSs, and PtPbY NPs for GOR electrocatalysis. Electrocatalytic GOR stability of PtPbY MNNs with (f) Comparisons of GLA selectivity and yield rate of PtPbY MNSs with reported electrocatalysts for GOR electrocatalysis. Electrocatalytic GOR stability of PtPbY MNNs with (g) different test times and (h) cycle numbers.

To further reveal why PtPbY MNSs hold the best performance for selective GLA electrocatalysis from GOR electrocatalysis, more electrocatalysts, including PtPb MNSs and PtPbY nanoparticles (NPs) were evaluated under the same conditions as their counterparts (Figs S27 and S28). Double-layer capacitance (C_dl_) experiments revealed a higher value of 1.83 mF cm^−2^ for PtPbY MNSs (Fig. 4a). In comparison, PtPb MNSs and PtPtY NPs showed remarkably lower C_dl_ values of 0.81 mF cm^−2^ and 0.64 mF cm^−2^, respectively. Obviously, both Y alloys and mesoporous structures synergistically enlarged electrochemical active sites for electrocatalysis (Fig. S29). Current density-potential profiles of GOR and water oxidation reaction (or oxygen evolution reaction, OER) were further presented (Fig. 4b). In the absence of glycerol, the current density was negligible in the potential range of 0∼1.05 V, indicating PtPbY MNSs were almost inactive for OER electrocatalysis. With the addition of 0.50 M glycerol, however, current density dramatically increased which was accompanied by a negative shift of onset potential to 0.37 V, indicating that glycerol was electrocatalytically oxidized by PtPbY MNSs. In comparison, PtPb MNSs and PtPbY NPs hold more positive onset potentials of 0.48 V and 0.49 V. Similarly, a higher current density of 159 mA cm^−2^ was achieved at 1.0 V for PtPbY MNSs, which reached 1.65 and 1.75 times higher than that of PtPb MNSs (97 mA cm^−2^) and PtPbY NPs (91 mA cm^−2^) (Fig. S30). The ECSA-normalized current densities also showed that PtPbY MNSs exhibited the highest specific activity of 0.65 mA cm_ECSA_^−2^ (Fig. S31). Moreover, PtPbY MNSs disclosed the lowest Tafel slope of 270 mV dec^−1^ for GOR electrocatalysis (Fig. 4c). The results thus highlighted the synergies of Y alloys and mesoporous structures of PtPbY MNSs to promote GOR electrocatalysis.

Electrocatalytic GOR selectivity and yield rate were then required for PtPbY MNSs, PtPb MNSs, and PtPbY NPs. As presented in Fig. 4d, PtPbY MNSs hold the highest GLA selectivity of 72.5%. Correspondingly, a remarkable sel._(GLA)_/sel._(others)_ value of 2.64 was achieved for PtPbY MNSs, which reached 2.81 and 1.82 times higher than that of PtPb MNSs and PtPbY NPs. Besides, a superior yield rate of 656 μmol mg_cat_^−1^ h^−1^ was obtained by PtPbY MNSs, which was remarkably higher than that of PtPb MNSs (316 μmol mg_cat_^−1^ h^−1^) and PtPbY NPs (298 μmol mg_cat_^−1^ h^−1^) (Fig. 4e). We also collected electrocatalytic GOR selectivity and yield rates under different test potentials (Fig. S32). With the increase of applied potentials, GLA selectivity from glycerol decreased slightly due to the competitive side reaction pathways and OER electrocatalysis. In comparison, yield rate of GLA showed a volcanic trend because of the increase of current density. When being further compared with state-of-the-art electrocatalysts reported in the literature, more impressively, PtPbY MNSs also represented the best choice for GLA electrosynthesis from glycerol (Fig. 4f and Table S1).

Electrocatalytic stability of PtPbY MNSs for selective GLA electrosynthesis from glycerol was conducted by two methods. First, chronoamperometric (CA) stability was evaluated by collecting GLA selectivity and productivity under different test times (Fig. 4g). With GOR electrocatalysis being tested for 10 h, GLA selectivity slightly declined from 72.5% to 62.0%, indicating GLA was not further electrooxidized into other products. Meanwhile, GLA productivity gradually increased from 656 μmol mg_cat_^−1^ to 3560 μmol mg_cat_^−1^, suggesting good electrocatalytic stability of PtPbY MNSs for GOR electrocatalysis. Physicochemical characterization of PtPbY MNSs after 10 h of CA test showed that the metal frameworks of PtPbY MNSs were well retained with only a slight loss of Y and partial oxidation of Pb (Figs S33 and S34). Second, cycling stability of PtPbY MNSs was assessed by continuously running CA tests (Fig. 4h). Impressively, there was almost no decline in both GLA selectivity and yield rate, even after being performed for 15 consecutive cycles.

**Figure 5.**
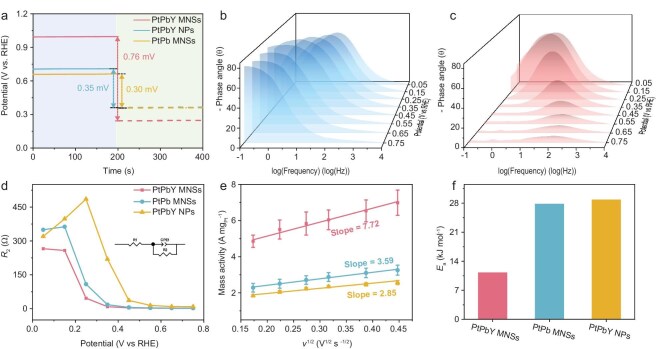
Electrochemical chemisorption and kinetics. (a) OCP values of PtPbY MNSs, PtPb MNSs, and PtPbY NPs collected in 1.0 M KOH with and without 0.50 M glycerol. Bode plots of PtPbY MNSs in 1.0 M KOH (b) without and (c) with 0.50 M glycerol. (d) Fitted *R_ct_* values of PtPbY MNSs, PtPb MNSs, and PtPbY NPs for GOR electrocatalysis. (e) The relationships between mass activity and *v*^1/2^ and (f) summarized *E_a_* values of PtPbY MNSs, PtPb MNSs, and PtPbY NPs.

Electrochemical kinetics were further evaluated to reveal the origin of superior GOR performance of PtPbY MNSs for GLA electrosynthesis. Considering glycerol and hydroxyl species as the primary reactants of GOR electrocatalysis, their competitive chemisorption properties were first studied in open-circuit potentials (OCPs) in 1.0 M KOH with and without glycerol (Δ*E*_OCP_). Impressively, PtPbY MNSs hold the highest Δ*E*_OCP_ value of 0.76 mV, confirming the strongest adsorption of glycerol (Fig. 5a). In comparison, PtPb MNSs and PtPbY NPs disclosed much lower Δ*E*_OCP_ values of 0.30 mV and 0.35 mV. Next, the adsorption capacity of hydroxyl species was evaluated using the Bode plots in 1.0 M KOH by *in situ* electrochemical impedance spectroscopy (*in situ* EIS). All three electrocatalysts hold characteristic peaks in the low-frequency region from 10^−1^ to 10^1^ Hz, which corresponded to the adsorption of hydroxyl species on the catalyst surface (Fig. 5b and Fig. S35) [56–58]. Compared with PtPb MNSs and PtPbY NPs, however, PtPbY MNSs disclosed a peak of higher intensity at 0.35 V–0.45 V, suggesting its enhanced ability to adsorb active hydroxyl species. Upon the addition of glycerol, the Bode plot peaks gradually shifted to the mid-frequency region (10^1^ to 10^4^ Hz) as the applied potentials were increased, which was accompanied by a rapid decrease in the phase angle from 0.25 V (Fig. 5c). The result indicated accelerated interfacial electron transfer, with GOR being predominant over the OER within the tested potentials. Meanwhile, in the mid-frequency region, the phase angle values of PtPbY MNSs were consistently lower than those of PtPb MNSs and PtPbY NPs across the entire applied potentials. Meanwhile, the phase angle of PtPbY MNSs decreased more rapidly, suggesting faster charge transfer kinetics and more efficient interfacial behavior for electrocatalysis. Besides, Nyquist plots and corresponding fitting parameters quantified the electrode interface reaction impedance, further demonstrating an accelerated interface charge transfer rate of PtPbY MNSs for electrocatalysis (Fig. 5d and Fig. S36).

Sweep rate-dependent tests and apparent activation energy (*E*_a_) of PtPbY MNSs were then conducted and further compared with PtPb MNSs and PtPbY NPs. Electrocatalytic performance was measured under different scan rates (*v*). Upon the increase of *v*, mass activities of all electrocatalysts increased remarkably (Fig. S37). We further correlated the relationship between mass activity and square root of *v* (*v*^1/2^) (Fig. 5e). All the electrocatalysts showed a nearly linear correlation, indicating that the kinetics of GOR electrocatalysis were governed by the diffusion. Notably, PtPbY MNSs exhibited a higher slope value of 7.7, suggesting the fastest diffusion kinetics for the reactants, intermediates, and products. Then, *E*_a_ values were calculated under different test temperatures according to the Arrhenius equation (k = Ae^−^*^E^*^a/RT^) (Fig. S38). In comparison to PtPb MNSs and PtPbY NPs, PtPbY MNSs endowed the lowest *E*_a_ value of 11.2 kJ mol^−1^, suggesting the smallest energy barrier for GOR electrocatalysis (Fig. 5f). The results definitively highlighted the importance of Y alloys and mesoporous structures in not only accelerating the adsorption of glycerol and the desorption of products but also promoting the reactivity of GOR electrocatalysis.

**Figure 6.**
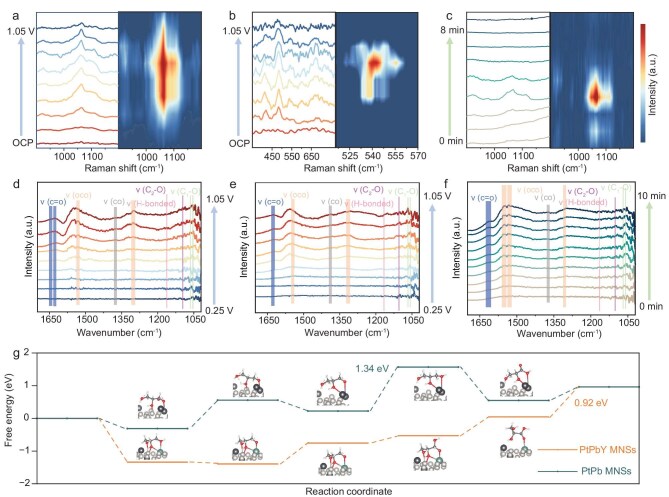
Electrocatalytic mechanism. (a) *In situ* Raman spectra and corresponding 2D images of PtPbY MNSs collected in different applied potentials at the Raman shifts of (a) 900–1200 cm^−1^ and (b) 400–700 cm^−1^. (c) *In situ* Raman spectra and corresponding 2D images of PtPbY MNSs collected at different test times at Raman shifts of 900–1200 cm^−1^. *In situ* FITR spectra of (d) PtPbY MNSs and (e) PtPb MNSs collected at different applied potentials. (f) *In situ* FITR spectra of PtPbY MNSs collected at different test times. (g) Comparison of free energy profiles of PtPbY and PtPb for GLA electrosynthesis from glycerol.

Further insights into chemisorption properties and corresponding reaction pathways were measured by *in situ* Raman and Fourier transform infrared spectroscopy (FTIR) measurements. Raman spectra were first performed on the catalyst surface (Fig. S39a). There were two characteristic υ_s_(C-OH) stretching peaks of glycerol in between 1017–1086 cm^−1^ and 1086–1134 cm^−1^. Interestingly, with applied potentials being increased from OCP to 1.05 V, the peak intensity of υ_s_(C-OH) stretching peaks gradually decreased and almost disappeared at 0.85 V, indicating the rapid adsorption of glycerol and further oxidation desorption of products on the catalyst surface (Fig. 6a) [18,59,60]. Correspondingly, there were peaks of C–C bond flexural vibrations of GLAD that appeared at 548 cm^−1^, whose intensities gradually weakened in the higher potentials (Fig. 6b). It suggested GLAD as the key intermediate for GLA electrosynthesis from GOR. In comparison, the peak intensities of GLAD collected in the electrolyte near the catalyst did not exhibit a significant change in all the applied potentials (Fig. S39b, c). The result confirmed that GLAD formed as the intermediate was further electrooxidized into GLA and would not be pulled out from the catalyst surface. Time-resolved Raman spectra further demonstrated that the intensities of the υ_s_(C-OH) stretching vibration peak of glycerol initially increased and then decreased, corresponding to the adsorption and accumulation of glycerol, followed by its oxidation and consumption on the catalyst surface (Fig. 6c and Fig. S40).

Dynamic chemisorption properties of PtPbY MNSs during GOR electrocatalysis were then measured by *in situ* FTIR spectra and further compared with PtPb MNSs. *In situ* Fourier transform infrared spectroscopy (FTIR) was then employed in the applied potentials from 0.25 V to 1.05 V (Fig. 6d, e). Both PtPbY MNSs and PtPb MNSs disclosed two series of adsorption bands at 1068 and 1054 cm^−1^ (primary ν(C_1_-O) stretching vibration) and 1152 cm^−1^ (secondary non-dissociated ν(C_2_-OH) vibration), indicating the adsorption of glycerol. With applied potentials being increased, ν(C=O) stretching vibration peaks at 1627 and 1644 cm^−1^, corresponding to GLAD, appeared, further indicating that GLAD served as an intermediate for GLA electrosynthesis [43,61,62]. There were distinct differences in the product distributions of PtPbY MNSs and PtPb MNSs. In comparison to PtPb MNSs, PtPbY MNSs hold remarkably enhanced intensities of GLA-related bands at 1300 cm⁻¹ and 1530 cm⁻¹, which suggested a higher efficiency in converting glycerol and GLAD to GLA. Similarly, there were weaker bonds at 1350 cm^−1^, indicating FA was not the main product for PtPbY MNSs. Moreover, time-resolved *in situ* FTIR spectra of PtPbY MNSs revealed a continuous increase in the concentration of GLA, while the content of formic acid remained negligible without significant increase with longer test times (Fig. 6f). In addition to the data from chemisorption properties of isopropanol and propanol (Fig. S41), our results highlighted optimized chemisorption properties of PtPbY MNSs for GLA electrosynthesis from glycerol, while minimizing undesirable side reactions.

DFT calculations were finally employed to compare the Gibbs free energy of GOR electrocatalysis for GLA electrosynthesis, including the adsorption/desorption of reaction intermediates, dehydrogenation, and hydroxyl insertion steps (Fig. 6g and Fig. S42). In comparison to PtPb, PtPbY with Y alloys showed a more negative adsorption energy for glycerol, indicating the stronger adsorption and enhanced activation of glycerol on the catalyst surface. For PtPbY, the rate-determining step (RDS) was identified as the desorption of GLA, with an energy barrier of 0.92 eV. By contrast, the RDS of PtPb was the hydroxyl attack step on GLAD, with a significantly higher energy barrier of 1.34 eV, which potentially facilitated the oxidation cleavage of C−C bonds for selective electrosynthesis of other C1 and C2 products. Meanwhile, Gibbs free energy of PtPbY was wholly smoother than that of PtPb MNSs, corresponding to the higher reactivity for GLA electrosynthesis. Besides, the charge-density analysis diagram on the surface of PtPbY showed that Y alloys activated the terminal C=O bond of GLAD thus facilitating the nucleophilic attack of hydroxyl species for selective GLA electrosynthesis (Fig. S43). Meanwhile, in comparison to PtPb MNSs, GLA was preferentially desorbed from PtPbY MNSs rather than further oxidation cleavage into C1/C2 products (Fig. S44). This aligned with DFT-predicted adsorption mode switching, where Y alloy shifted the bidentate-O coordination to monodentate carbonyl-O binding on PtPbY MNSs, which thus facilitated the desorption and electrosynthesis of GLA with high selectivity and production.

## CONCLUSION

In conclusion, Y alloys were demonstrated as an efficient route to optimize chemisorption properties of 2D PtPb MNSs for selective GLA electrosynthesis from glycerol in an ambient condition. A series of REM-alloyed PtPb MNSs were prepared by a two-step template-free method, which thus endowed abundant mesopores, quasi-single-crystalline structures, surface-clean metal sites, and tunable REMs for electrocatalysis. When being used as electrocatalyst for GOR, PtPbY MNSs disclosed remarkable performance, including GLA selectivity of 72.5%, superior yield rate of 656 μmol mg_cat_^−1^ h^−1^, and high stability for >15 cycles, and represented one of the best electrocatalysts for GLA electrosynthesis. Mechanism studies revealed that the synergies of Y alloys and mesoporous structures not only kinetically accelerated the adsorption of glycerol and its electrooxidation reaction but also favored the desorption of GLA. In particular, the Y atom not only enhanced the adsorption of reactant molecules, reducing the energy barrier for the glycerol electro-oxidation reaction to synthesize GLA by electronic/bifunctional effects (Fig. S46), but also altered the adsorption mode of GLA on the catalyst surface, lowering the desorption energy barrier, inhibiting deep oxidation, and improving the selectivity of GLA by geometric effects. This work provides a facile yet sustainable strategy to prepare novel mesoporous metal electrocatalysts with optimized chemisorption properties by utilizing REM alloys for selective electrosynthesis of high value-added chemicals from various wastes.

## Supplementary Material

nwaf343_Supplemental_File
