# Supplementary material for: Efficient glyceric acid electrosynthesis from waste glycerol on rare-earth-metal-alloyed mesoporous PtPb nanosheets
Source: Natl Sci Rev. 2025 Aug 19;12(10):nwaf343. doi: 10.1093/nsr/nwaf343 (PMC12485611; doi:10.1093/nsr/nwaf343)
Supplement: nwaf343_Supplemental_File [file nwaf343_supplemental_file.pdf]

Supplementary Data for

Efficient glyceric acid electrosynthesis from waste glycerol on rare-earth-metal-alloyed mesoporous PtPb nanosheets

Dongping Fan[1], Lizhi Sun[1], Ruijia Yuan[1], Zhen-An Qiao[2], Shunai Che[3,*], and Ben Liu[1,*]

[1]Key Laboratory of Green Chemistry and Technology of Ministry of Education, National and Local Joint Engineering Laboratory of Energy Plant Bio-fuel Preparation and Utilization, College of Chemistry, Sichuan University, Chengdu 610064, China. E-mail: ben.liu@scu.edu.cn

[2]State Key Laboratory of Inorganic Synthesis and Preparative Chemistry, Jilin University, Changchun 130012, China

[3]School of Chemistry and Chemical Engineering, Frontiers Science Center for Transformative Molecules, State Key Laboratory of Composite Materials, Shanghai Key Laboratory for Molecular Engineering of Chiral Drugs, Shanghai Jiao Tong University, Shanghai 200240, China. Emai: chesa@sjtu.edu.cn

Methods

Materials and Chemicals

Platinum(II) acetylacetonate ($Pt(acac)_2$, ≥98%), lead(II) acetylacetonate ($Pb(acac)_2$, ≥98%), lanthanum(III) acetylacetonate hydrate ($La(acac)_3 \cdot xH_2O$, ≥99.9%), cerium(III) acetylacetonate hydrate ($Ce(acac)_3 \cdot xH_2O$, ≥99%), praseodymium (III) acetylacetonate hydrate ($Pr(acac)_3 \cdot xH_2O$, ≥99%), neodymium (III) acetylacetonate ($Nd(acac)_3$, ≥98%), samarium(III) acetylacetonate hydrate ($Sm(acac)_3 \cdot xH_2O$, ≥98%), europium(III) acetylacetonate hydrate ($Eu(acac)_3 \cdot xH_2O$, ≥99.9%), gadolinium(III) acetylacetonate hydrate ($Gd(acac)_3 \cdot xH_2O$, ≥99.9%), terbium(III) acetylacetonate ($Tb(acac)_3$, ≥98%), dysprosium(III) acetylacetonate ($Dy(acac)_3$, ≥99.9%), erbium (III) acetylacetonate ($Er(acac)_3$, ≥99%), thulium(III) acetylacetonate ($Tm(acac)_3$, ≥99.99%), Ytterbium (III) acetylacetonate hydrate ($Yb(acac)_3 \cdot xH_2O$, ≥99%), Lutetium(III) acetylacetonate ($Lu(acac)_3$, ≥99%), yttrium(III) acetylacetonate hydrate ($Y(acac)_3 \cdot xH_2O$, ≥99%), ($CH_3(CH_2)_7CH=CH(CH_2)_7CH_2NH_2$, OAm, ≥90%), 1-octadecene ($CH_2=CH(CH_2)_{15}CH_3$, ODE, ≥90%), cyclohexane (C_6H_{12}, 99.9%), glycidaldehyde ($C_3H_6O_3$, 90%), 1,3-dihydroxyacetone ($C_3H_6O_3$, 98%), lactic acid ($C_3H_6O_3$, 90%), glyceric acid ($C_3H_6O_4$, 20% in water), tartronic acid ($C_3H_4O_5$, 98%), deuterium oxide (D_2O, 99.9%), and maleic acid ($C_4H_4O_4$, 99%) were purchased from *Adamas-beta. L*-ascorbic acid (AA, ≥99%), potassium hydroxide (KOH, ≥90.0%), ethanol (CH_3CH_2OH, ≥99.8%), formic acid (HCOOH, 98%), nitric acid (HNO_3, 65.0%-68.0%), sulfuric acid (H_2SO_4, 95.0%-98.0%), glycerol ($C_3H_8O_3$, 98%) and acetic acid (CH_3COOH, ≥99.5%) were obtained from Sinopharm Chemical Reagent Co. Ltd. (Shanghai, China). Commercial Pt nanoparticles (NPs) catalyst (20 wt%) was purchased from Sigma-Aldrich. Nafion solution (5 wt.%) was obtained from Macklin. All the reagents were used as received without further purification. The deionized H_2O used in all experiments was with the resistivity of 18.25 mΩ.

Synthesis of REM-alloyed PtPb and PtPb intermetallic nanosheets

REM-alloyed PtPb intermetallic nanosheets were synthesized with a simple solvothermal method with a modified route reported in the literature (*Science* **2016**, *354*, 1410). Take PtPbY INSs as an example, typically, 16.0 mg of $Pt(acac)_2$, 16.0 mg of $Pb(acac)_2$, and 4.0 mg of $Yb(acac)_3 \cdot xH_2O$ were dissolved in a mixture consisting of 5.0 mL of octadecene and 5.0 mL of oleamine and formed homogeneity after stirring for 8 h at room temperature. Then, 73 mg of AA was added to the mixture, further heated from room temperature to 160 ^{o}C under magnetic stirring condition, and kept another 6 h. Finally, the product was obtained after being washed and centrifugated with cyclohexane for several times. Similarly, synthesis process of other REM-alloyed PtPb and PtPb intermetallic nanosheets was the same as the above process but with different precursor of REMs or without REM. Meanwhile, the ratios of Y were well adjustable by changing the feed ratios of precursors.

Synthesis of REM-alloyed PtPb MNSs

REM-alloyed PtPb MNSs were prepared by selective etching Pb atoms of REM-alloyed PtPb intermetallic nanosheets as parent template. Typically, 10 mg of as-synthesized REM-alloyed PtPb intermetallic nanosheets were etched by 10 mL of HNO_3, where the volume ratio of HNO_3 and H_2O was 2:1. After being reacted for 2h, REM-alloyed PtPb MNSs with abundant mesopores were obtained after being centrifugated and washed for three times with ethanol/cyclohexane.

Synthesis of PtPbY NPs

PtPbY NPs were synthesized by a simple solvothermal method, where 16.0 mg of $Pt(acac)_2$, 2.0 mg of $Pb(acac)_2$, and 4.0 mg of $Y(acac)_3$ were mixed and dissolved in 2.5 mL of OAm and 2.5 mL of ODE. After being stirred for 8 h, the mixture was heated to 160 °C and allowed to react for an additional 6 h. PtPbY NPs were ultimately collected through being centrifuged and washed for three times with ethanol/cyclohexane.

Preparation of electrocatalyst ink

5.0 mg of catalyst and 2.5 mg of carbon were dispersed in 1.0 mL of a dispersant containing ethanol, water, and Nafion, with a volume ratio of 0.70 mL : 0.25 mL : 0.05 mL for ethanol : water : Nafion. After being sonicated for 30 min, 50 μL of catalyst ink was drop-cast onto a 1.0 cm^{-2} carbon paper surface, resulting in a catalyst loading of 0.25 mg. Finally, after being dried at room temperature, the sample was ready for use as a working electrode for electrocatalytic measurements.

Electrocatalytic GOR measurements

Electrocatalytic GOR measurements were conducted using a CHI 660E electrochemical workstation in a single-compartment electrolytic cell at a constant room temperature of 25 °C. A three-electrodes system was used for all electrochemical tests, where Hg/HgO, carbon rod, and carbon loaded with catalyst were used as the reference electrode, the counter electrode, and the working electrode, respectively. For all electrochemical tests in this work, KOH (1.0 M) with and without glycerol (0.50 M) were used as the electrolyte, and the electrode potentials were converted to the reversible hydrogen electrode (RHE) according to the following equation (1):

$$E_{\mathrm{RHE}} = E_{\mathrm{Hg/HgO}} + 0.098\ \mathrm{V} + 0.0591(\mathrm{pH}) \quad (1)$$

Linear sweep voltammetry (LSV) measurements were carried out at a scan rate of 10 mV s^{-1} within a potential range of 0.024 to 1.124 V until the polarization curves reached a steady state. Chronoamperometry measurements were conducted at various applied potentials for 1.0 h in a different applied potential varying from 0.55 V to 1.05 V, with the solution stirred at a constant rate of 800 rpm. Cyclic voltammetry (CV) curves in electrochemical double-layer capacitance (C_{dl}) determinations were measured in a potential window nearly without the Faradaic process at different scan rates of 40, 60, 80, 100, 120, 140, and 160 mV s^{-1}.

Products analysis of GOR electrocatalysis

The products analysis was performed by the proton nuclear magnetic spectroscopy (^1H NMR) and quantified by the external standard calibration curves. Typically, the electrolyte was collected through chronoamperometric tests and detected by ^1H NMR spectrometer. ^1H spectra were recorded on an AVANCE NEO 400 instruments, in which 500 µL of electrolyte was mixed with 50 µL of D_2O with maleic acid as an internal standard.

The selectivity (%) of products could be calculated as the following equitation (2):

$$\text{Selectivity (\%)} = n_{\text{product}}/n_{\text{total product}} * 100\% \tag{2}$$

where $n_{\text{total product}}$ (mol) was the productivity of total products.

The yield rate (µmol mg_{cat}^{-1} h^{-1}) of product could be calculated using the following equation (3):

$$\text{Yield rate (product)} = (C_{\text{product}} \times V)/(t \times m) \tag{3}$$

where C_{product} was the measured product concentration (µmol mL^{-1}); V was the volume of the electrolyte (10 mL); t was the electrolysis time (1 h); m was the mass of the catalysts (mg).

Characterization

Microscopic features of catalysts were performed by using a field emission transmission electron microscope (JEM-F200, JEOL Ltd., Japan) with an accelerating voltage of 200 kV. The crystalline phase was characterized by Powder X-ray diffraction (XRD) patterns using a D/max 2500 VL/PC diffractometer (Japan) equipped with graphite-monochromatized Cu Kα radiation in 2ϑ ranging from 30° to 90°. The working voltage and current were 40 kV and 100 mA, respectively. Inductively coupled plasma mass spectrometry was recorded on a NexION 350D. In situ Raman measurements were performed on a Renishaw inVia-Qontor with a 785 nm laser as excitation source and a CHI 660E electrochemical workstation. The cell was made by Teflon shell with a quartz window between the sample and objective. The Ag/AgCl electrode, carbon rod and carbon loaded with catalysts served as reference, counter electrodes and working electrodes, respectively. In situ FTIR spectra

were recorded by a Thermo Scientific Nicolet IS50 with MCT detector, between 4000 and 1000 cm^{-1} with a resolution of 0.005 cm^{-1}. Electrochemical measurements were recorded on a CHI 660E electrochemical workstation in a three-electrode configuration at room temperature, using Ag/AgCl electrode and carbon rod as reference and counter electrode, respectively.

Computational Details

All density functional theory (DFT) periodic calculations were conducted using the Vienna Ab-initio Simulations Package (VASP)[1,2]. The Perdew-Burke-Ernzerhof (PBE) functional was utilized, and interactions were modeled with the projector-augmented wave (PAW) potential. The Kohn-Sham one-electron valence states were expanded using a plane wave basis with a 450 eV cutoff energy[3-5].

The vacuum thickness was maintained at over 15 Å to effectively separate the two opposing surface effects. To manage computational costs, Monkhorst-Pack reciprocal space integration was performed using Gamma-centered k-points with a mesh of 3 × 3 × 1. The convergence criterion for the Hellmann-Feynman forces was established at less than 0.1 eV/Å. The electronic energy for both structural optimization and frequency calculations was set to less than 10^{-5} eV.

The d-band center was used to understand the adsorption capacity of the different catalyst surface. The calculation formula of d-band center[6]:

$$\varepsilon_d = \frac{\int_{-\infty}^{\infty} n_d(\varepsilon)\varepsilon \mathrm{d}\varepsilon}{\int_{-\infty}^{\infty} n_d(\varepsilon)\mathrm{d}\varepsilon}$$

The Gibbs free energy also was computed in this study. The zero-point energy (ZPE) correction was applied following previously reported approaches. In the DFT process, we calculated the Gibbs free energy using the following equations:

$$G^0 = \mathrm{E}_{DFT} + ZPE - TS^0$$

Where G^0 is the Gibbs free energy, E_{DFT} is total free energy, ZPE is the vibration energy; TS^0 is the entropy change (T = 298.15 K).

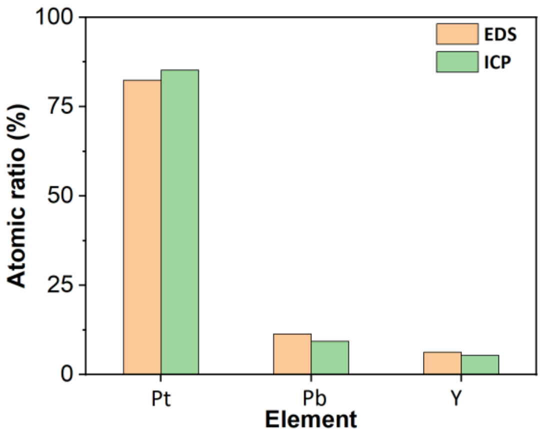

Figure S1. Atomic ratios of PtPbY MNSs collected from STEM EDS mapping images and inductively coupled plasma mass spectrometry.

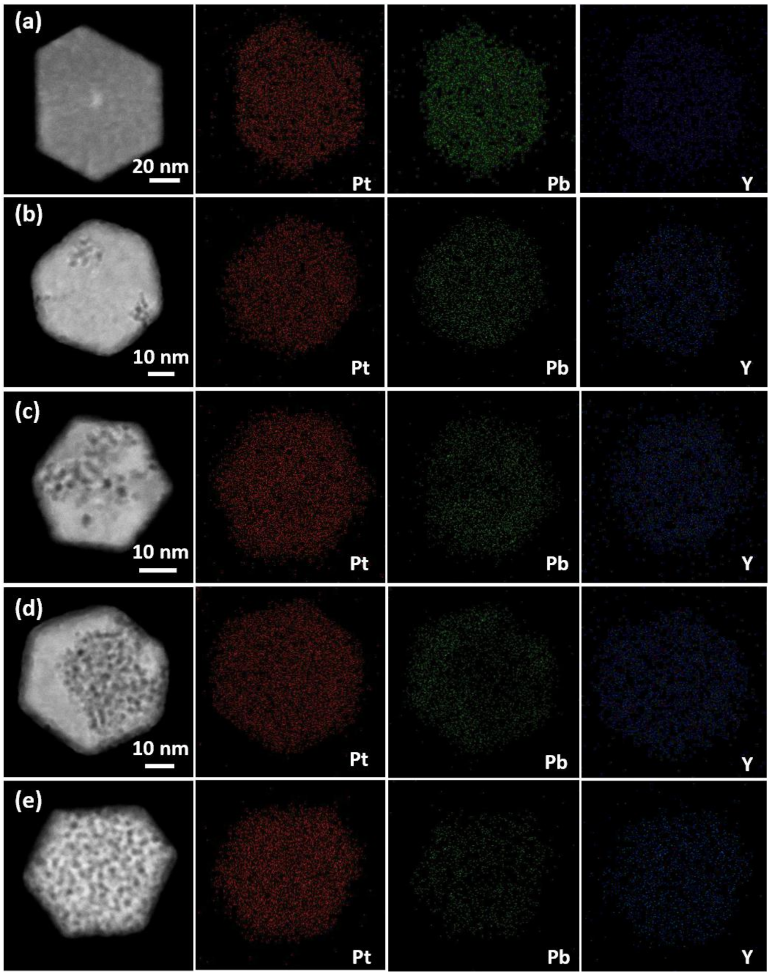

Figure S2. HAADF-STEM images and corresponding EDX mapping images of PtPbY MNSs synthesized with different etching times of (a) 0 min, (b) 5 min, (c) 30 min, (d) 60 min, and (e) 120 min.

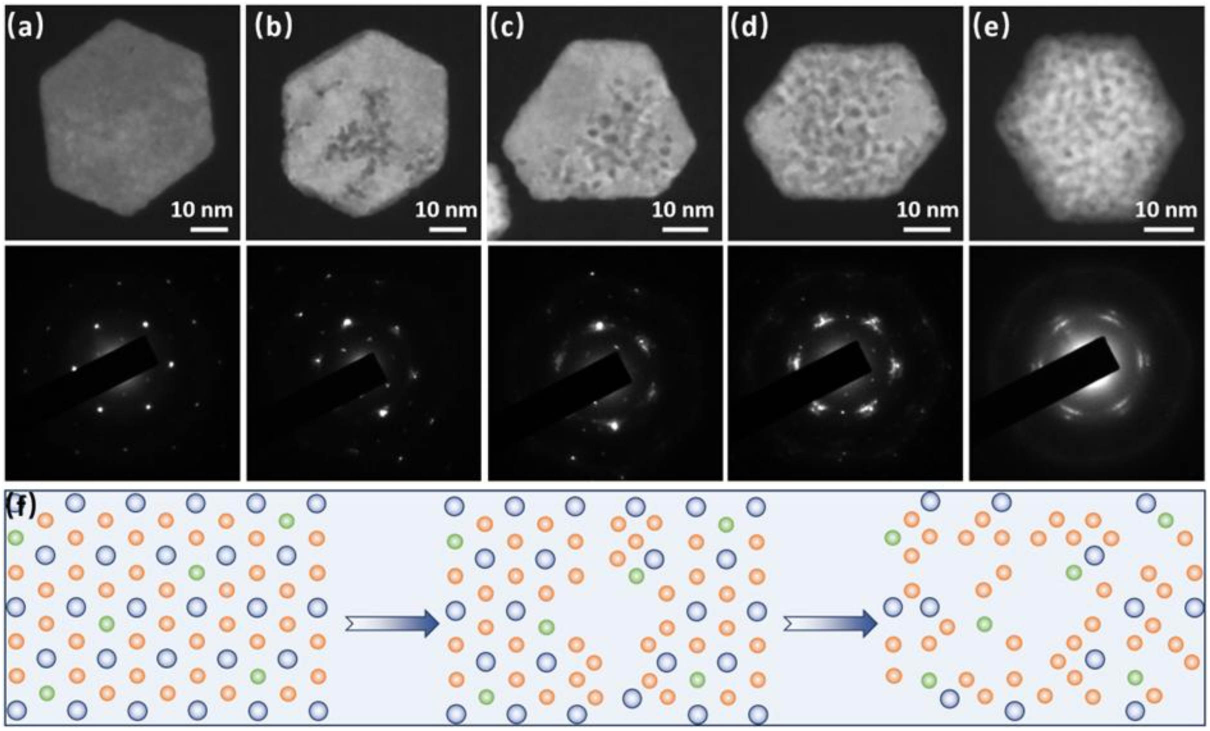

Figure S3. HAADF-STEM images and corresponding SAED patterns of PtPbY MNSs synthesized with different etching times of (a) 0 min, (b) 5 min, (c) 30 min, (d) 60 min, and (e) 120 min. (f) Proposed formation process of 2D PtPbY MNPs.

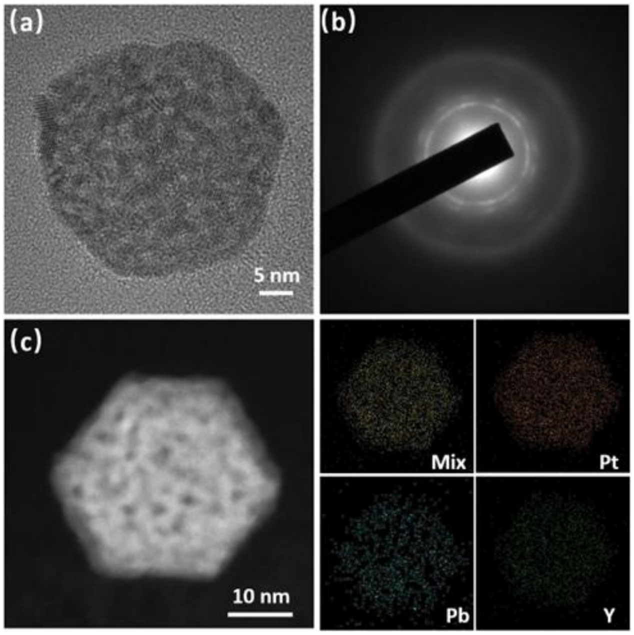

Figure S4. (a) TEM image, (b) SAED pattern, (c) STEM and EDS mapping images of PtPbY MNSs obtained by adopting the rapid etching with strong acid.

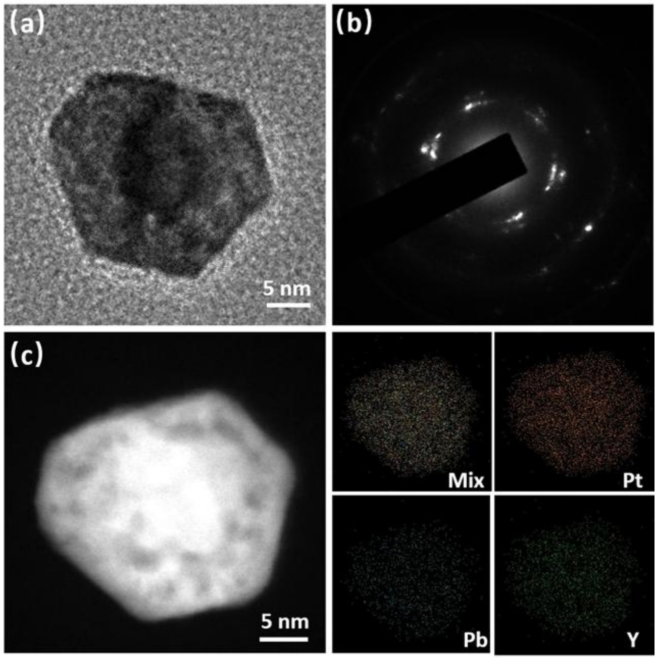

Figure S5. (a) TEM image, (b) SAED pattern, (c) STEM and EDS mapping images of PtPbY MNSs obtained by using the PtPbY alloy (non-intermetallic compound) as parent template.

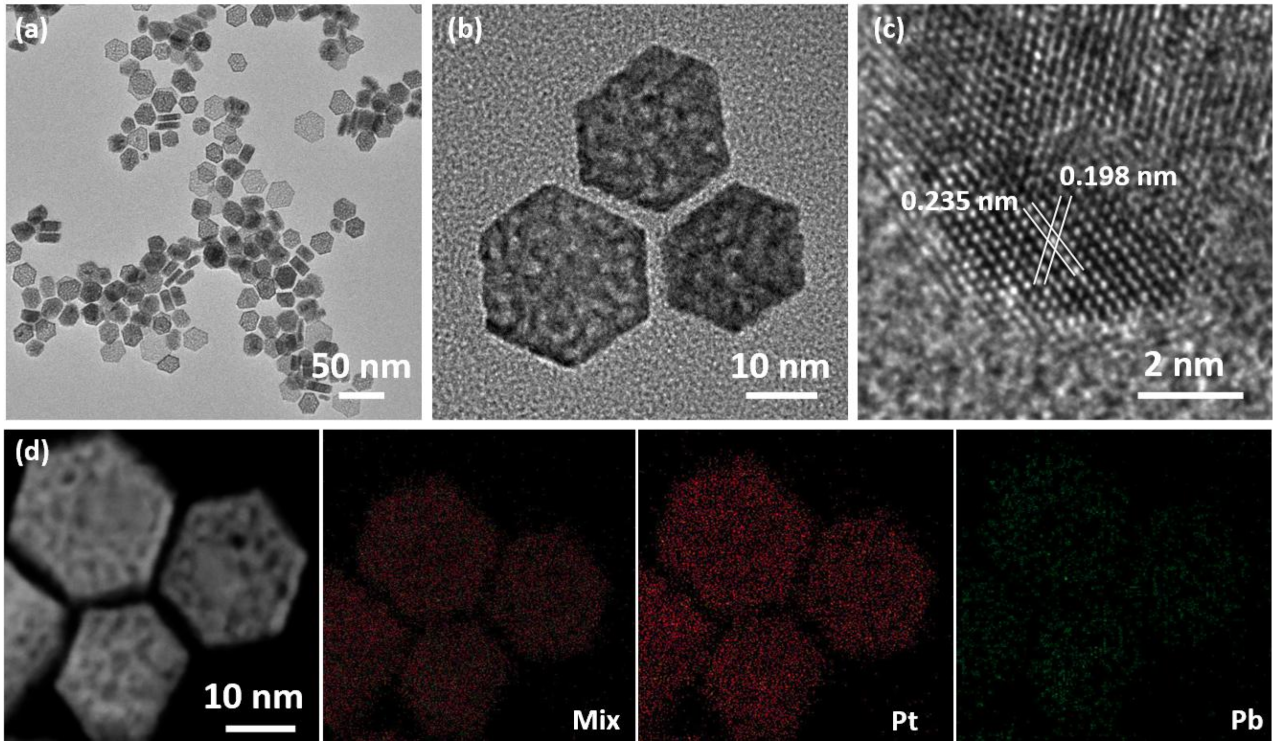

Figure S6. (a) Low-magnification and (b) high-magnification TEM images, (c) high-resolution TEM image, (d) HAADF-STEM image and corresponding EDX mapping images of PtPb MNSs.

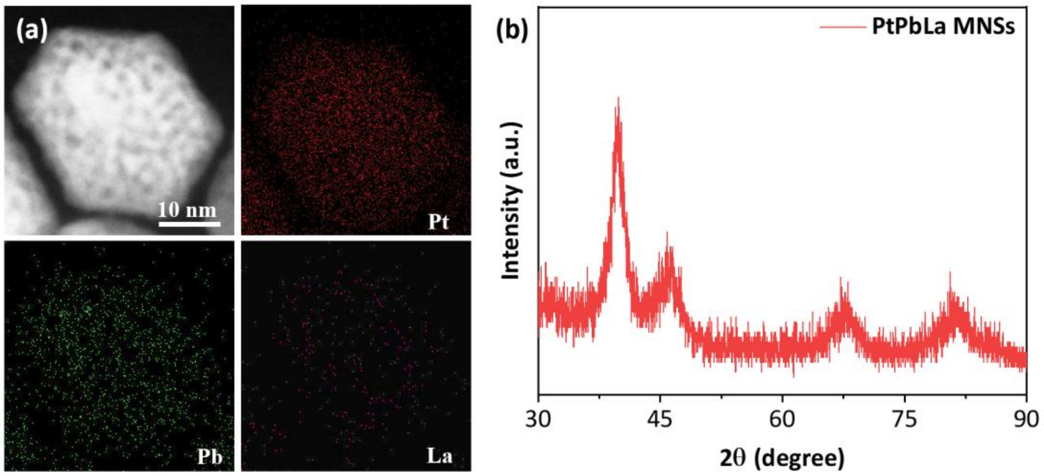

Figure S7. (a) HAADF-STEM image and corresponding EDX mapping images, and (b) powder XRD pattern of PtPbLa MNSs.

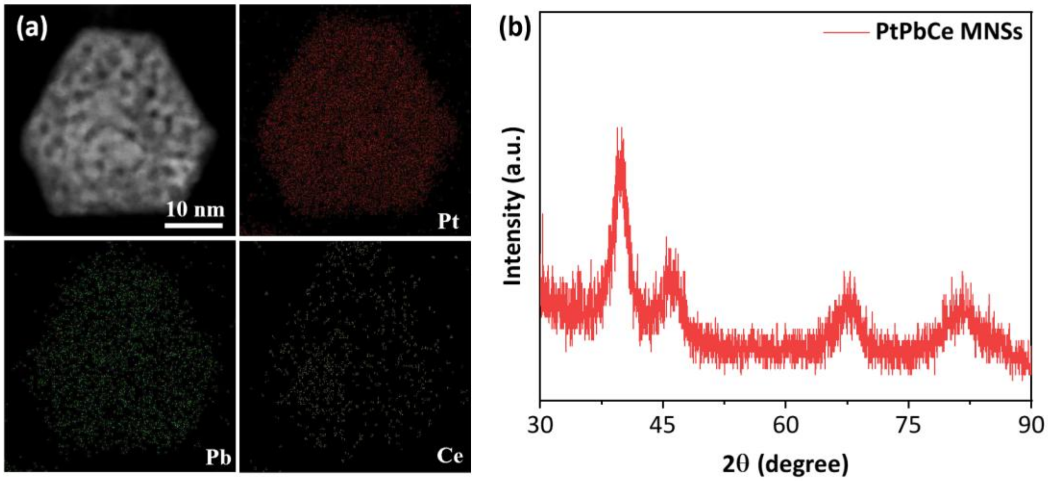

Figure S8. (a) HAADF-STEM image and corresponding EDX mapping images, and (b) powder XRD pattern of PtPbCe MNSs.

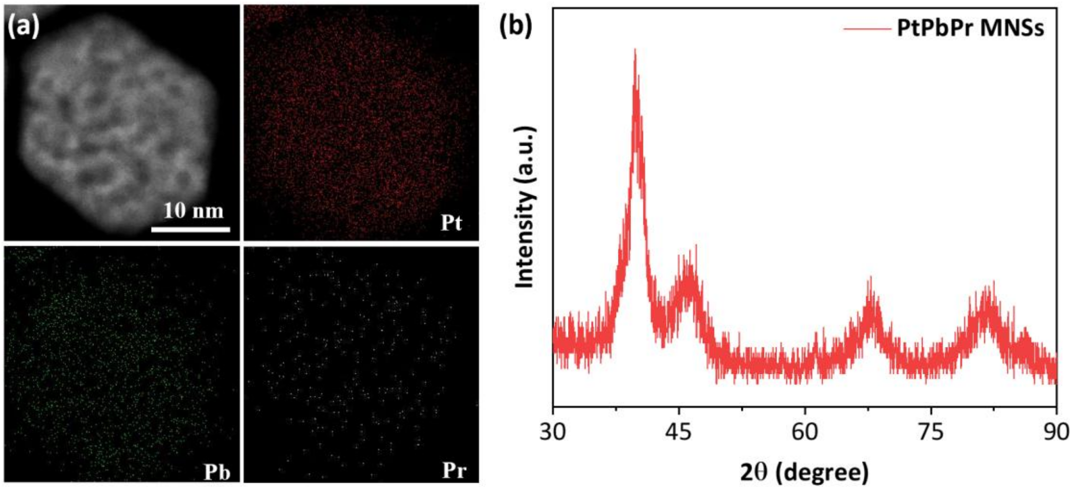

Figure S9. (a) HAADF-STEM image and corresponding EDX mapping images, and (b) powder XRD pattern of PtPbPr MNSs.

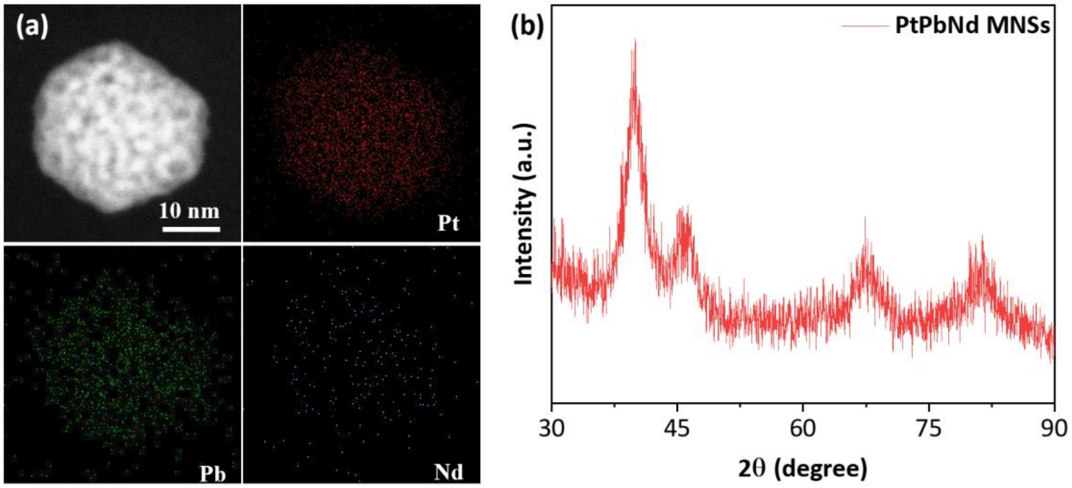

Figure S10. (a) HAADF-STEM image and corresponding EDX mapping images, and (b) powder XRD pattern of PtPbNd MNSs.

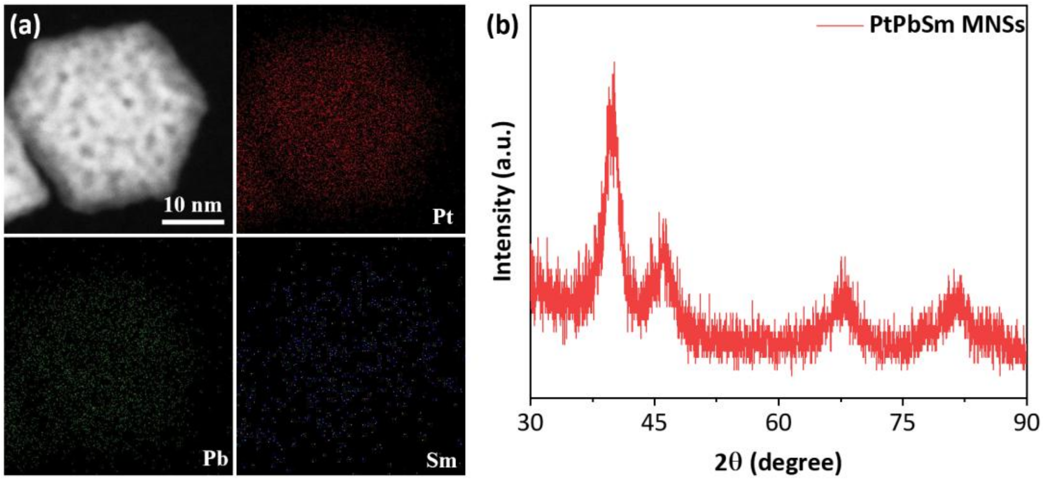

Figure S11. (a) HAADF-STEM image and corresponding EDX mapping images, and (b) powder XRD pattern of PtPbSm MNSs.

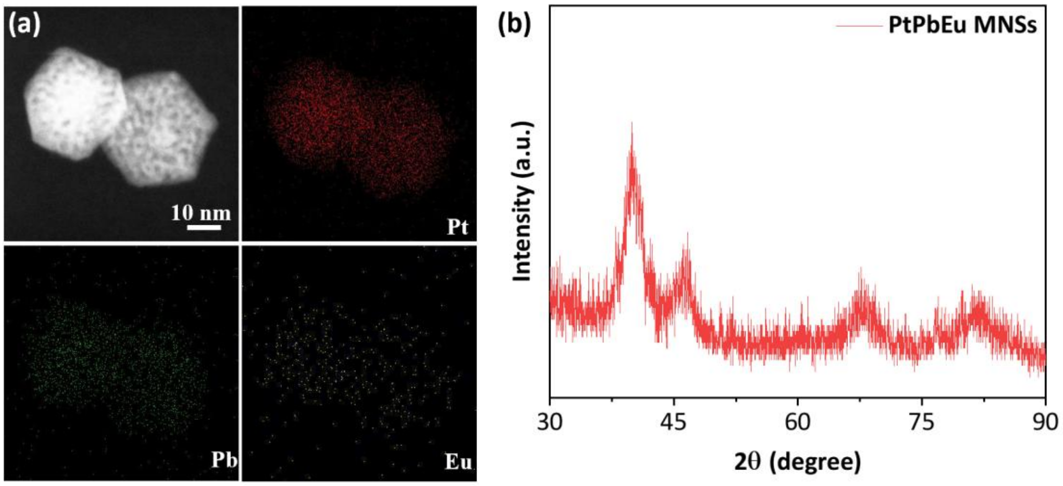

Figure S12. (a) HAADF-STEM image and corresponding EDX mapping images, and (b) powder XRD pattern of PtPbEu MNSs.

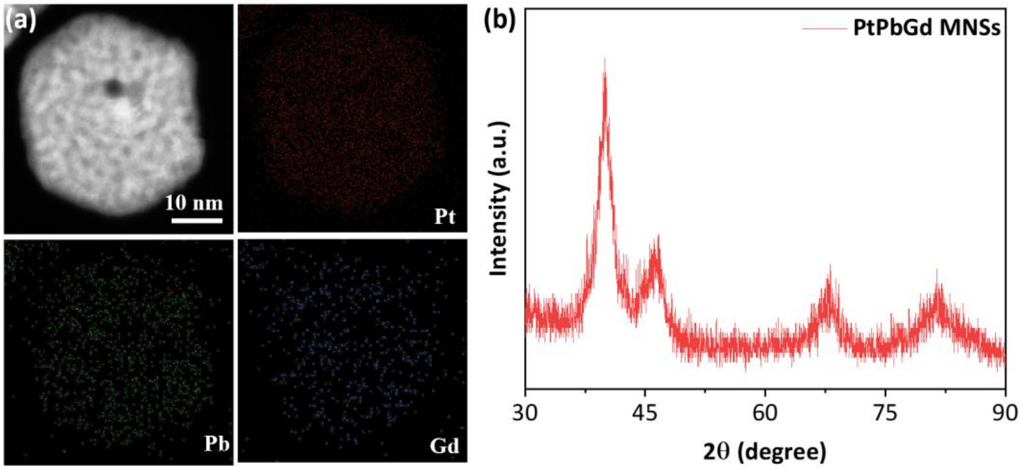

Figure S13. (a) HAADF-STEM image and corresponding EDX mapping images, and (b) powder XRD pattern of PtPbGd MNSs.

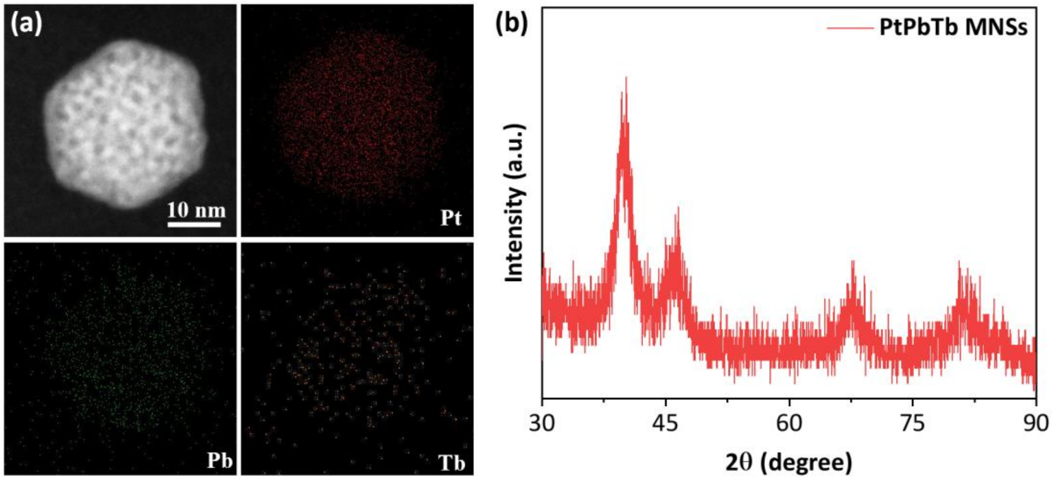

Figure S14. (a) HAADF-STEM image and corresponding EDX mapping images, and (b) powder XRD pattern of PtPbTb MNSs.

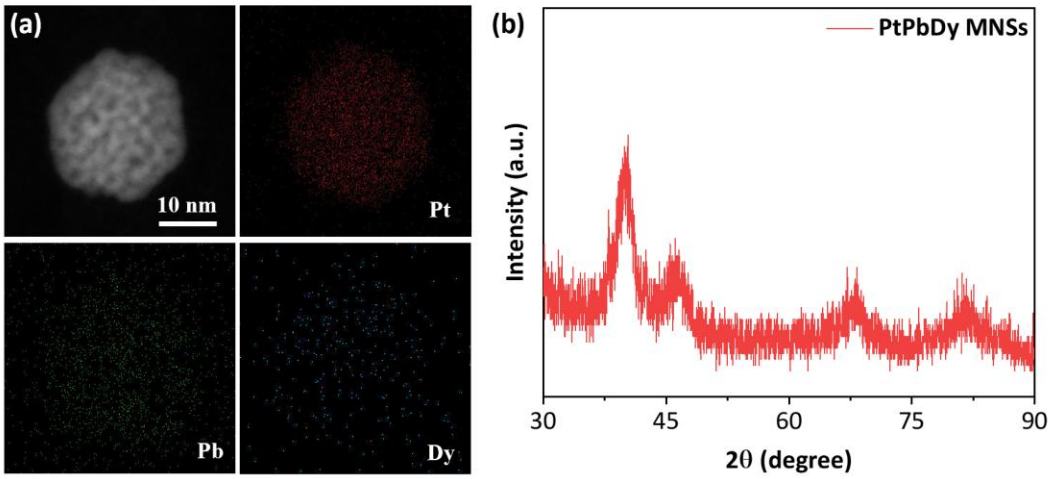

Figure S15. (a) HAADF-STEM image and corresponding EDX mapping images, and (b) powder XRD pattern of PtPbDy MNSs.

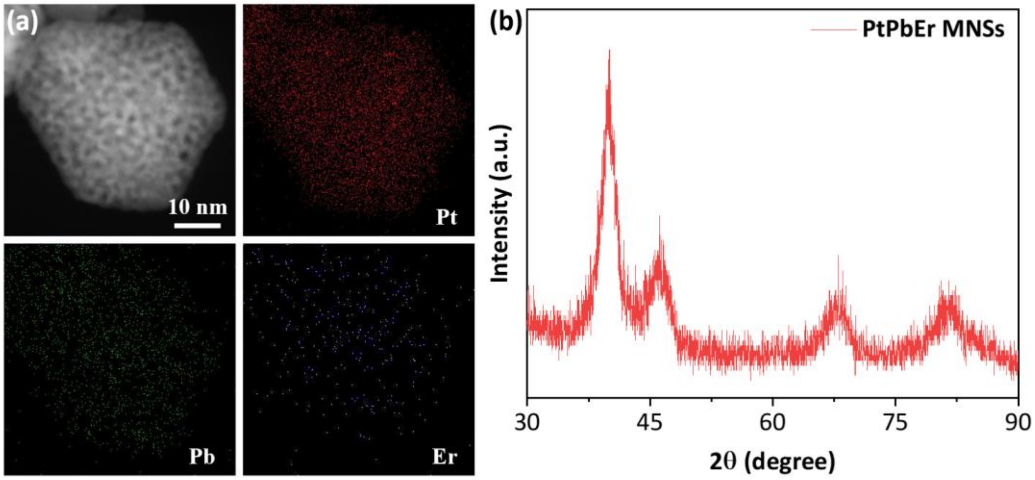

Figure S16. (a) HAADF-STEM image and corresponding EDX mapping images, and (b) powder XRD pattern of PtPbEr MNSs.

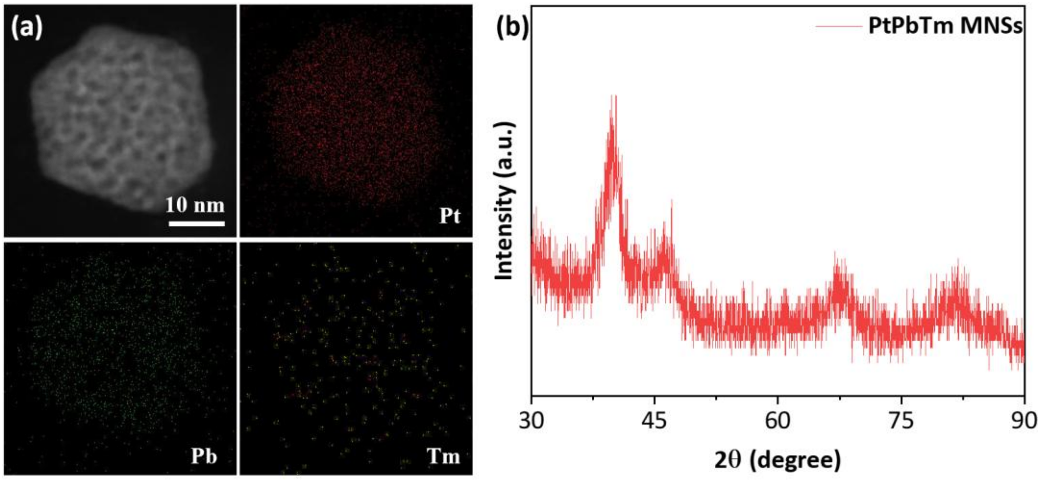

Figure S17. (a) HAADF-STEM image and corresponding EDX mapping images, and (b) powder XRD pattern of PtPbTm MNSs.

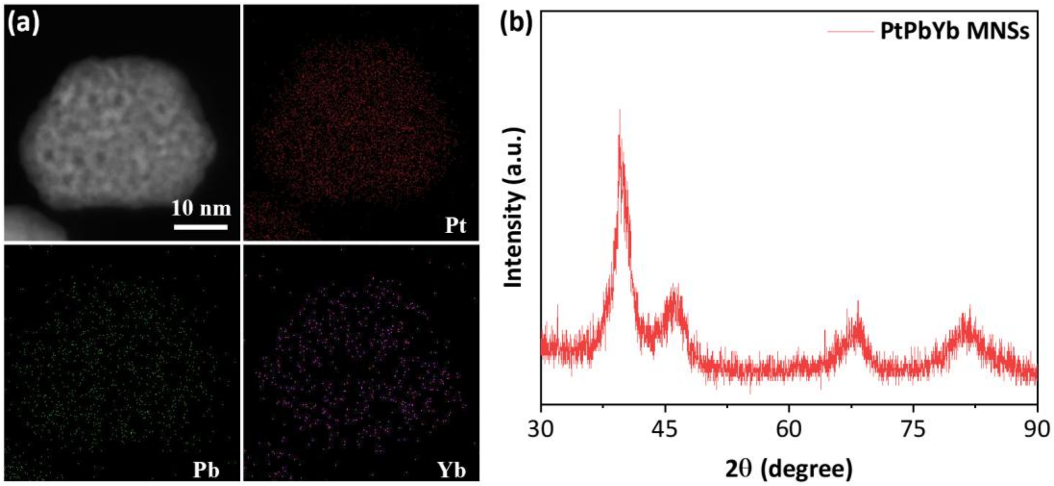

Figure S18. (a) HAADF-STEM image and corresponding EDX mapping images, and (b) powder XRD pattern of PtPbYb MNSs.

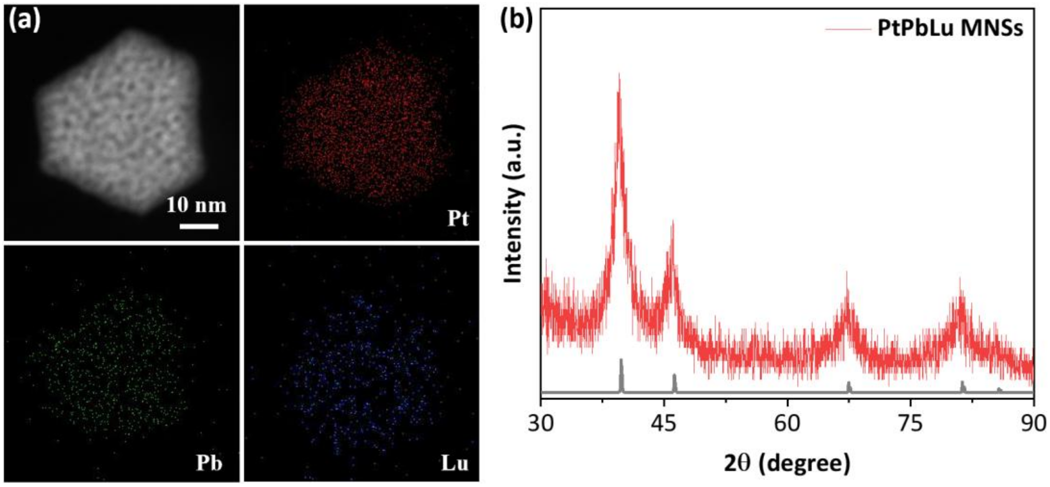

Figure S19. (a) HAADF-STEM image and corresponding EDX mapping images, and (b) powder XRD pattern of PtPbLu MNSs.

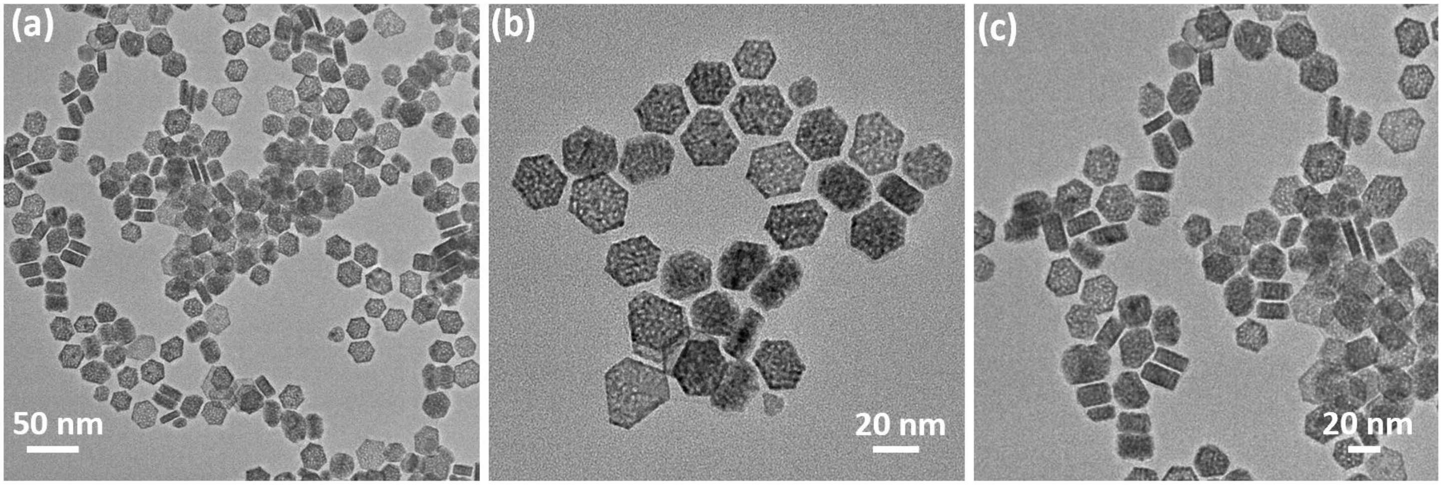

Figure S20. (a-c) More low-magnification and high-magnification TEM images of PtPbY MNSs.

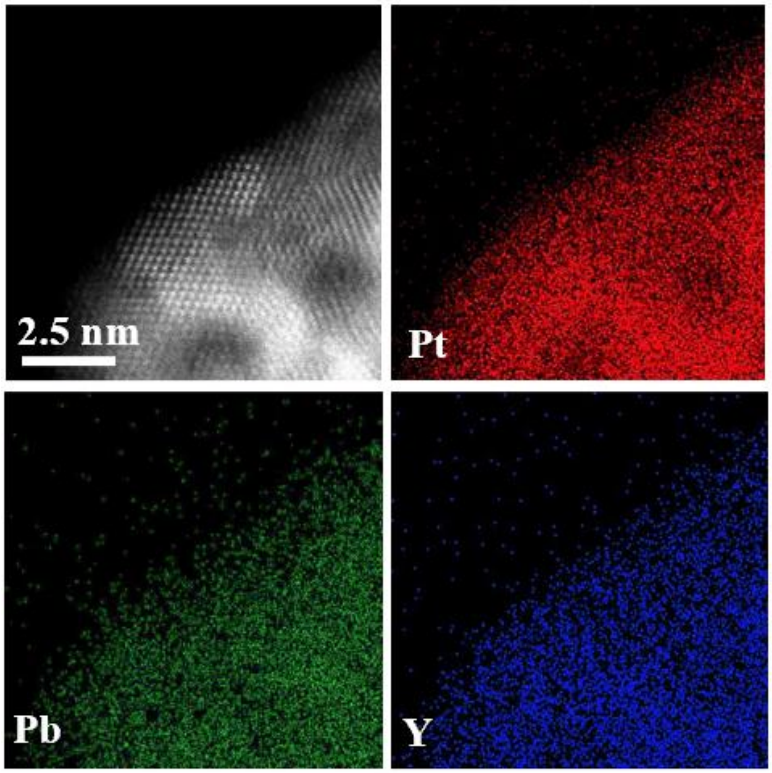

Figure S21. HAADF-STEM image and corresponding EDX mapping images of PtPbY MNSs observed along the amplified edges.

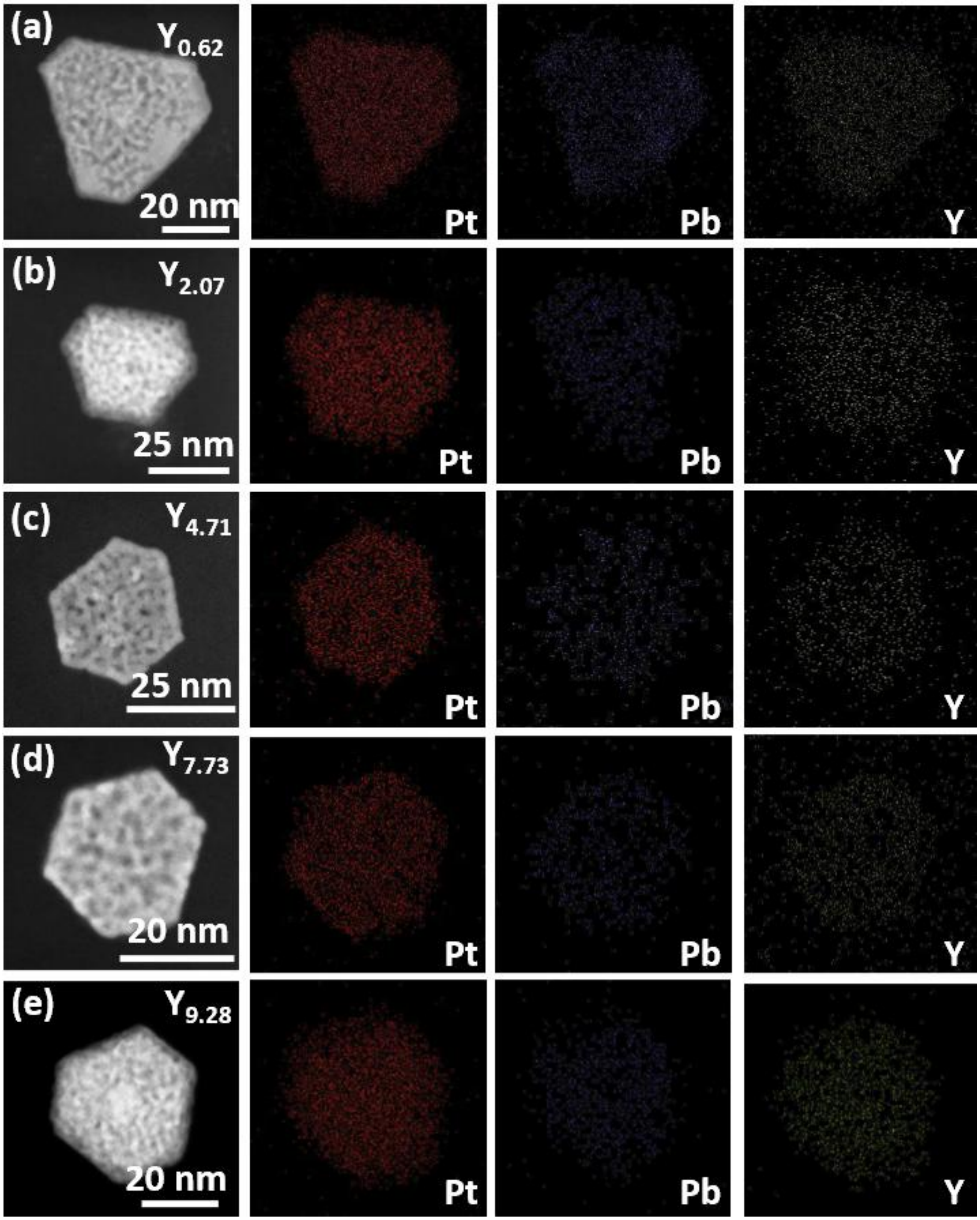

Figure S22. HAADF-STEM image and corresponding EDX mapping images of PtPbY MNSs with different Y ratios of (a) 0.62%, (b) 2.07 %, (c) 4.71%, (d) 7.73%, and (e) 9.28%.

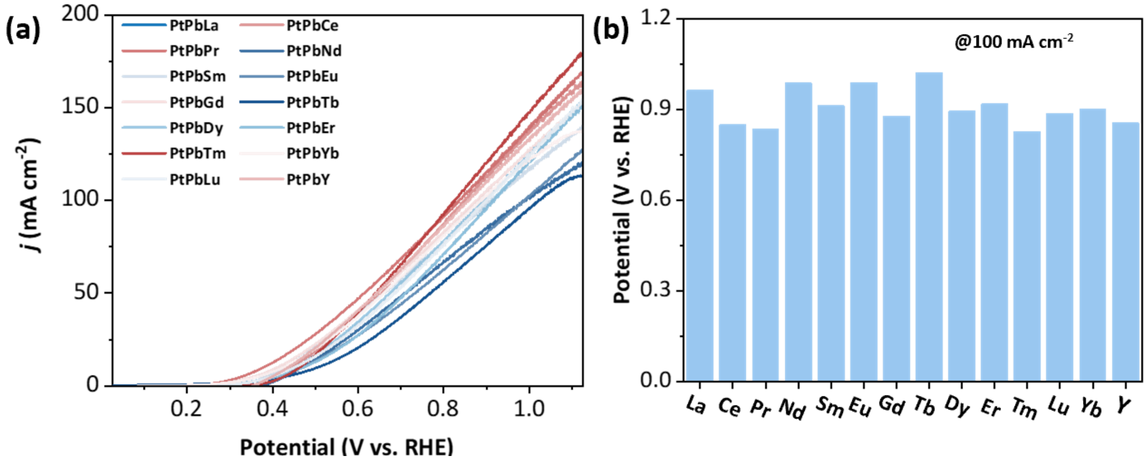

Figure S23. (a) LSV curves and (b) summarized overpotentials (at the current density of 100 mA cm^{-2}) of 14 REM-alloyed PtPb MNSs for GOR.

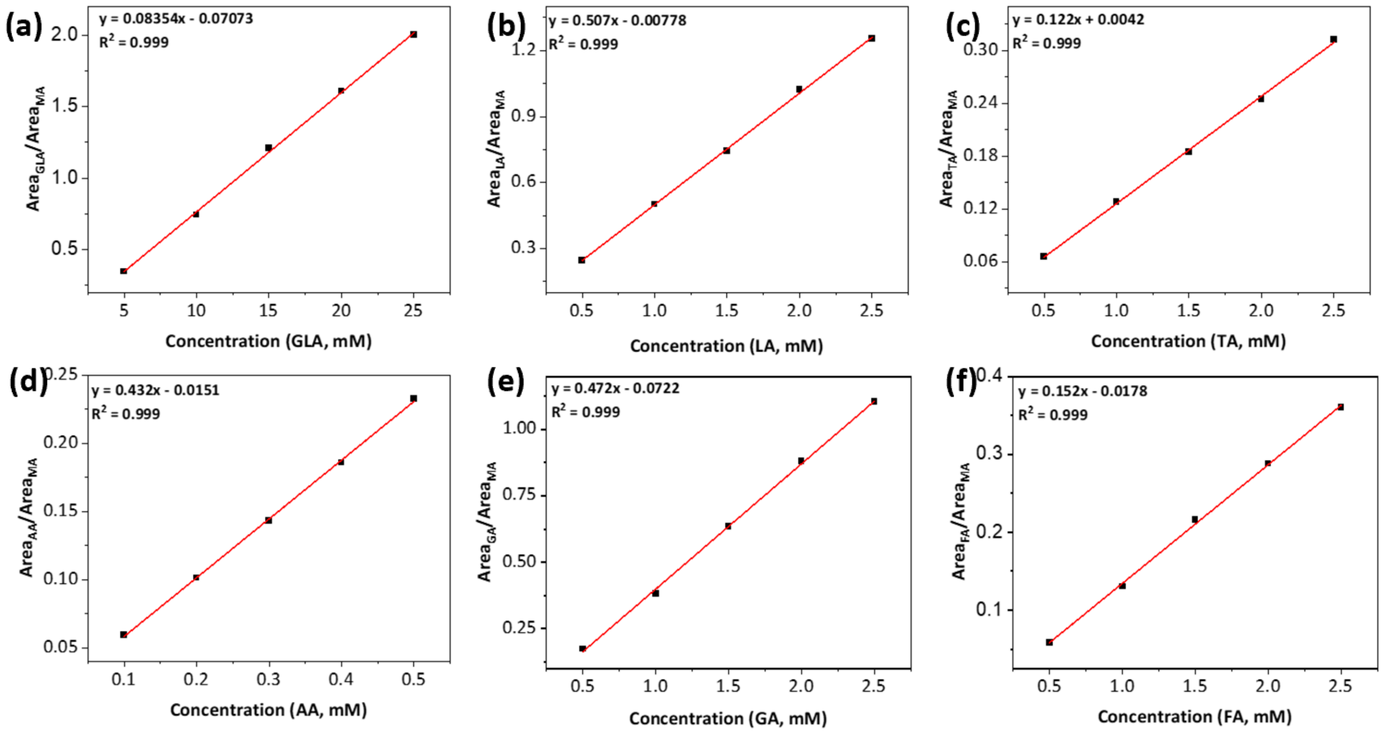

Figure S24. External standard calibration curves of (a) GLA, (b) LA, (c) TA, (d) AA, (e) GA, and (f) FA by proton nuclear magnetic spectroscopy (^1H NMR).

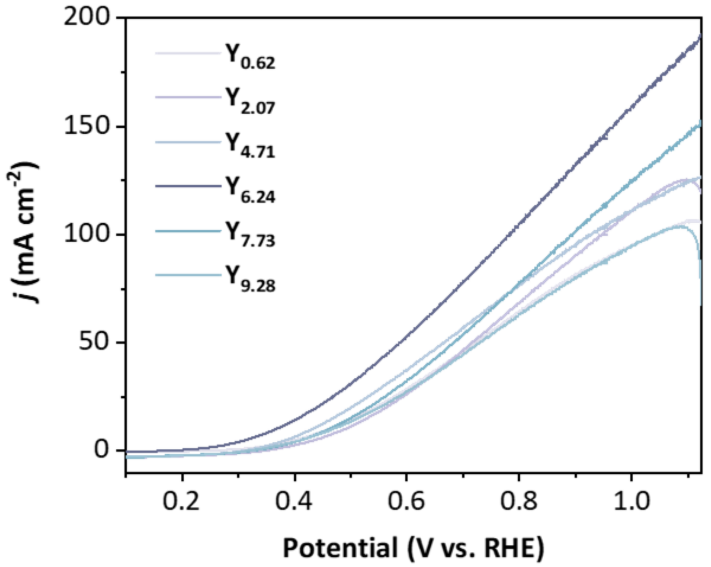

Figure S25. LSV curves of PtPbY MNSs with different Y ratios.

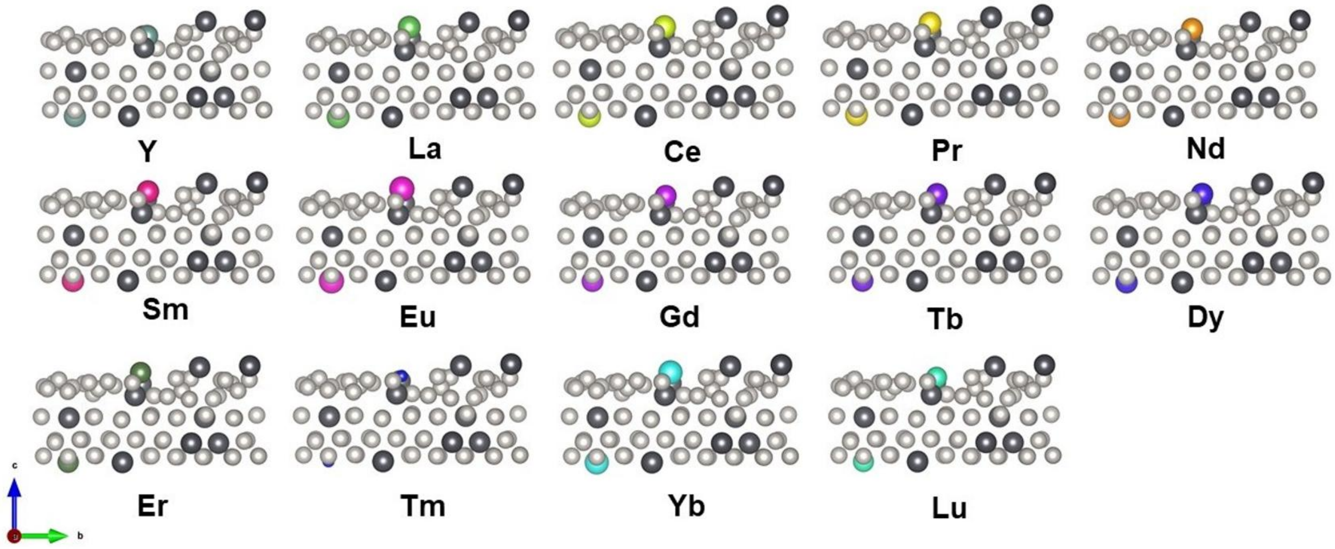

Figure S26. Side views of optimized models of REM elementals of PtPb MNSs (Y、La、Ce、Pr、Nd、Sm、Eu、Gd、Tb、Dy、Er、Tm、Yb、Lu).

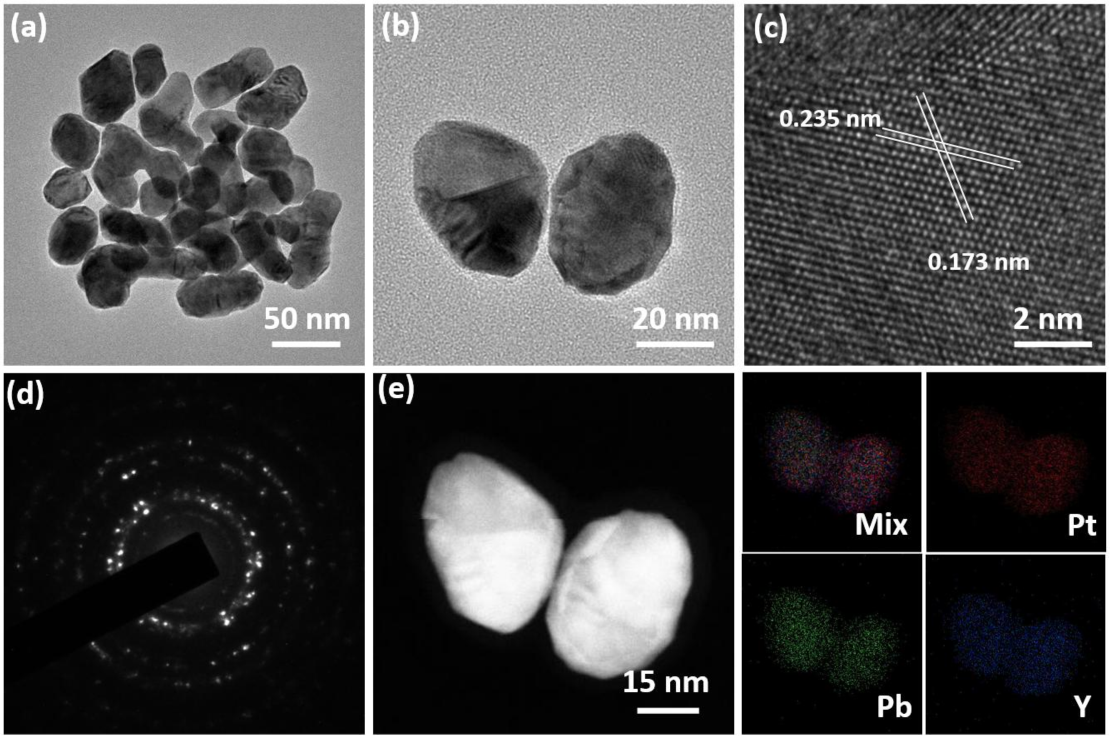

Figure S27. (a) Low-magnification and (b) high-magnification TEM images, (c) high-resolution TEM image, (d) SAED pattern, and (e) HAADF-STEM image and corresponding EDX mapping images of PtPbY NPs.

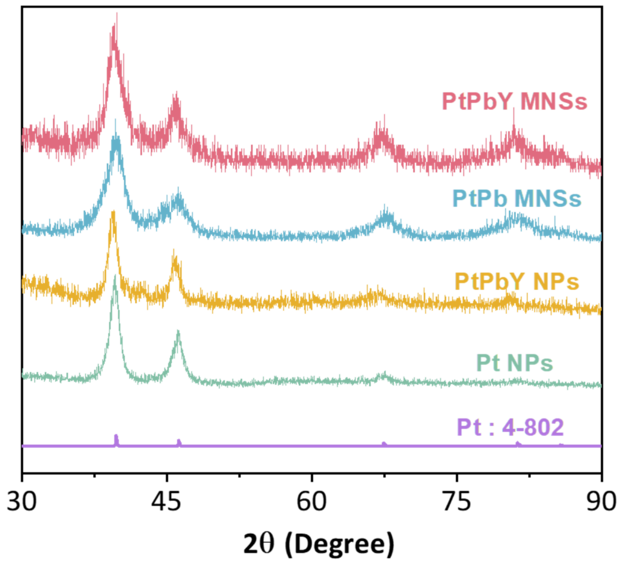

Figure S28. Powder XRD patterns of PtPbY MNSs, PtPb MNSs, and PtPbY NPs.

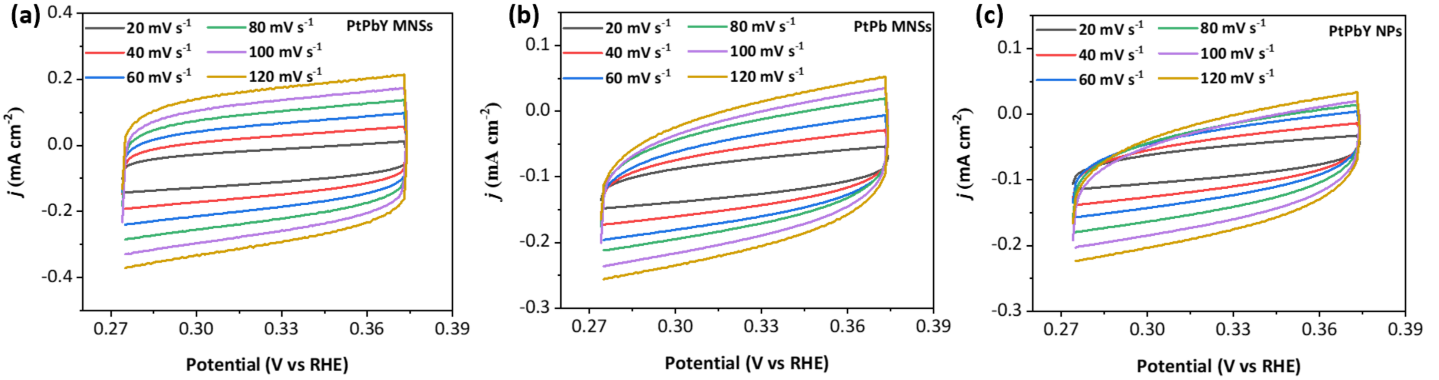

Figure S29. CV curves of (a) PtPbY MNSs, (b) PtPb MNSs, and (c) PtPbY NPs collected in non-faradic regions at different scan rates.

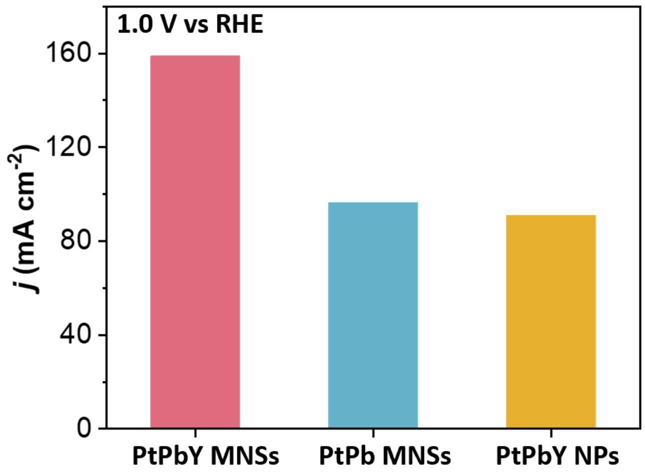

Figure S30. Summarized current densities of PtPbY MNSs, PtPb MNSs, and PtPbY NPs collected at applied potential of 1.0 V.

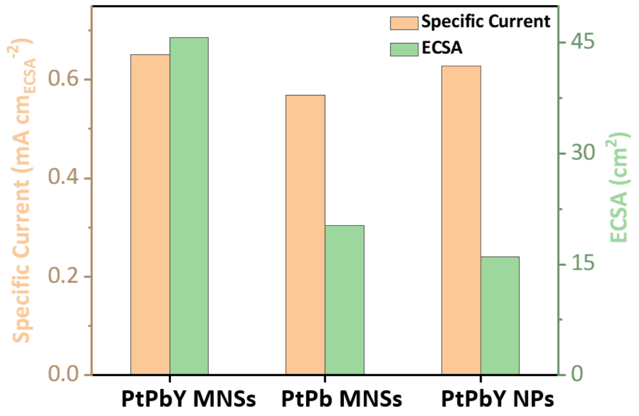

Figure S31. Summarized ECSA and ECSA-normalized current densities of PtPbY MNSs, PtPb MNSs, and PtPbY NPs collected at applied potential of η_{10} for PtPbY NPs.

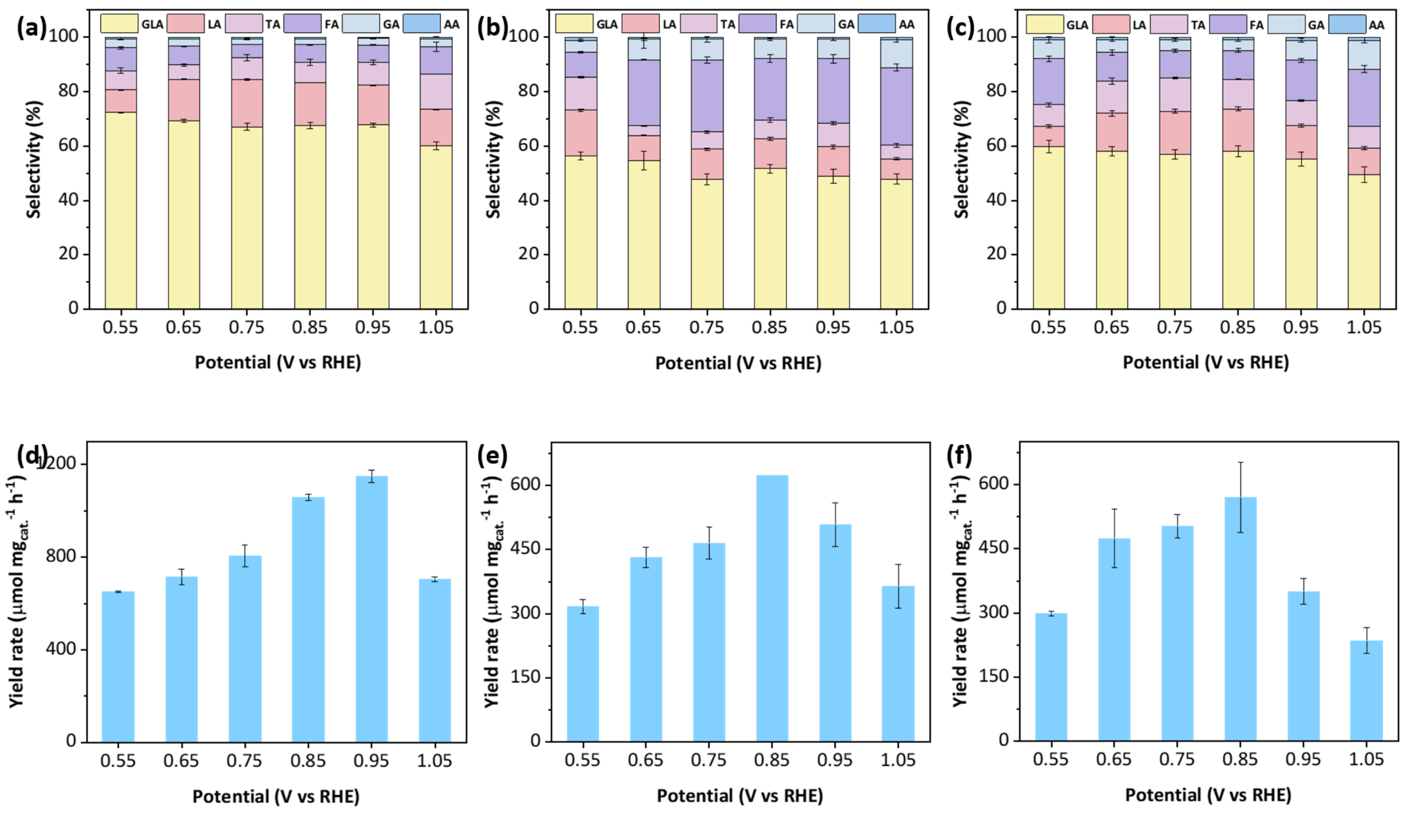

Figure S32. (a-c) GLA selectivity and (d-f) yield rate of PtPbY MNSs, PtPb MNSs, and PtPbY NPs for GOR electrocatalysis.

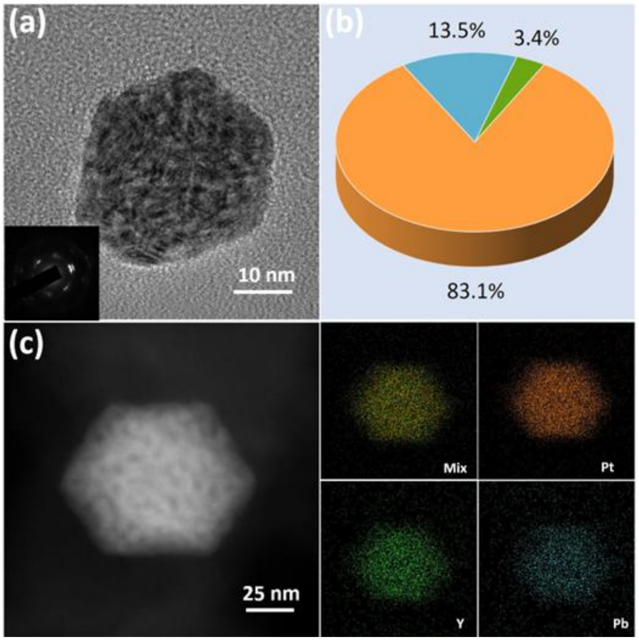

Figure S33. (a) TEM image and corresponding SAED pattern (inset), (b) atomic ratio analysis, and (c) HAADF-STEM image and corresponding EDX mapping images of PtPbY MNSs after 10 h CA stability test.

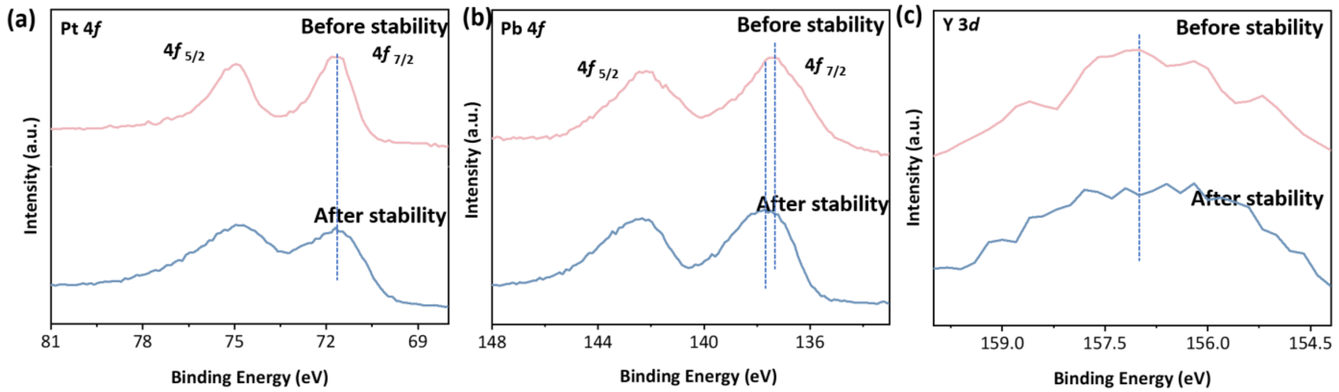

Figure S34. High-resolution XPS spectra for (a) Pt 4f, (b) Pb 4f, and (c) Y 3d of PtPbY MNSs before and after the stability test.

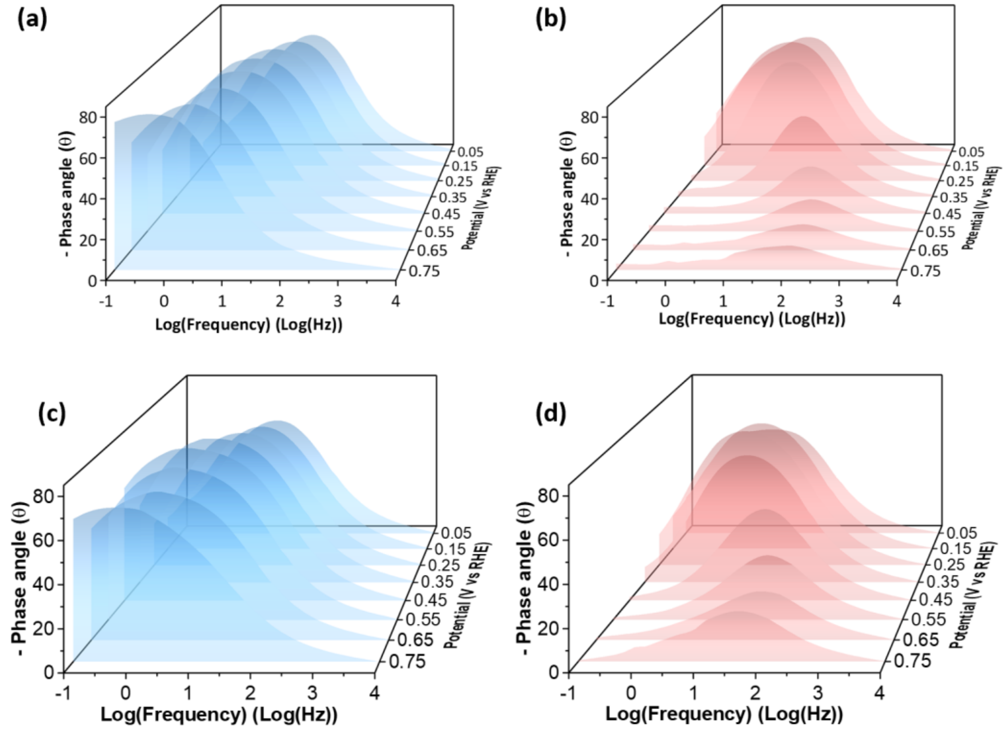

Figure S35. Bode plots of (a,b) PtPb MNSs and (c,d) PtPbY NPs collected in (a,c) 1.0 M KOH and (b,d) 1.0 M KOH + 0.50 M glycerol.

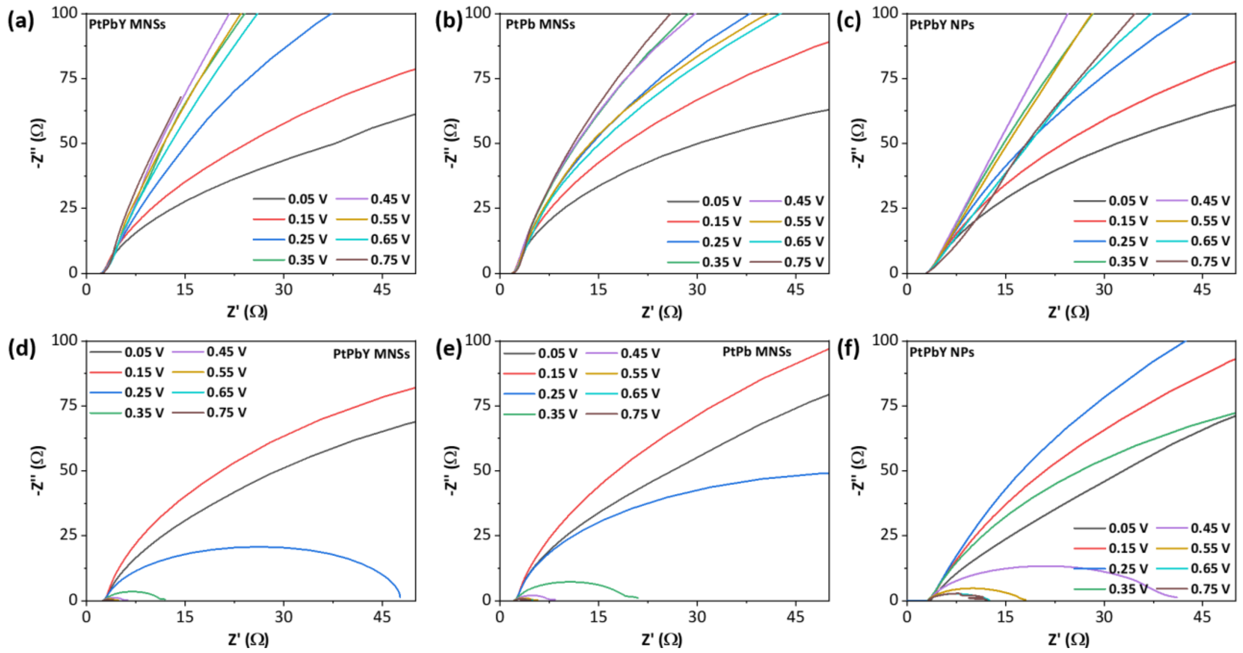

Figure S36. Nyquist plots of (a,d) PtPbY MNSs, (b,e) PtPb MNSs, and (c,f) PtPbY NPs in (a,b,c) 1.0 M KOH and (d,e,f) 1.0 M KOH + 0.50 M glycerol.

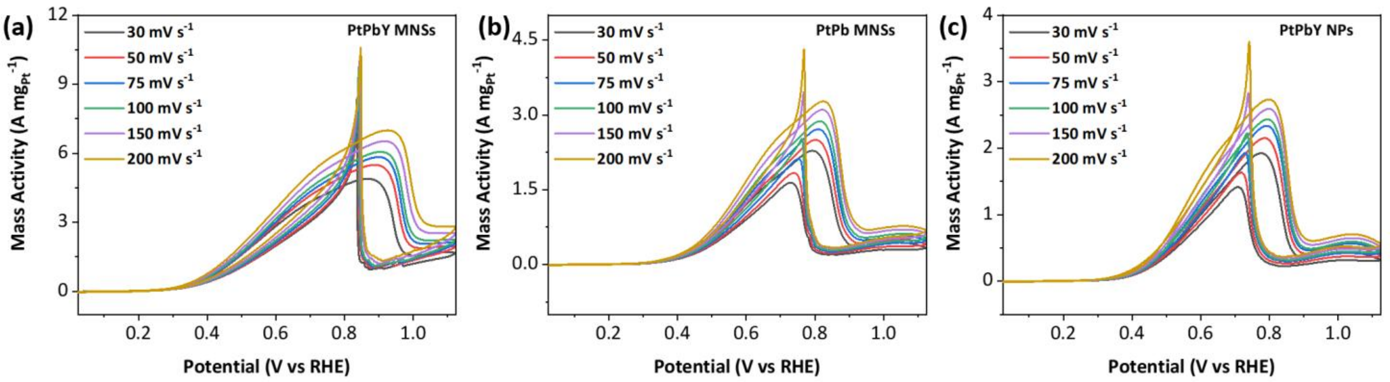

Figure S37. CV curves of (a) PtPbY MNSs, (b) PtPb MNSs, and (c) PtPbY NP collected with different scan rates in 1.0 M KOH + 0.50 M glycerol.

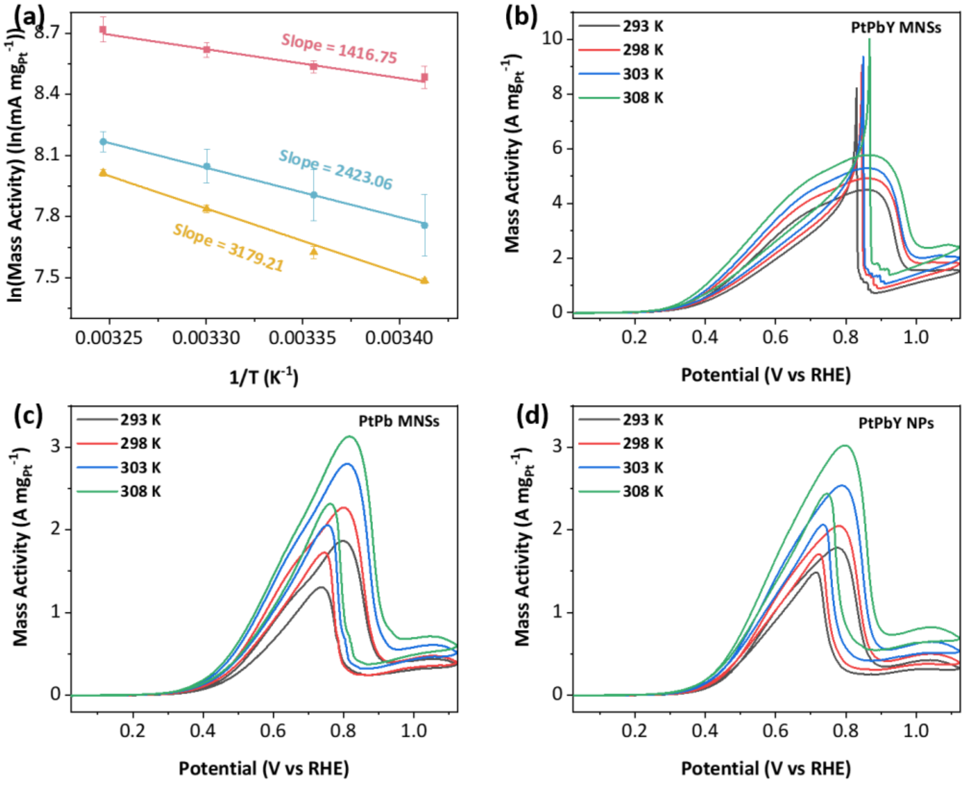

Figure S38. (a) Summarized linear relationships between ln(mass activity) and 1/T. CV curves of (b) PtPbY MNSs, (c) PtPb MNSs, and (d) PtPbY NPs collected with different reaction temperatures in 1.0 M KOH + 0.50 M glycerol.

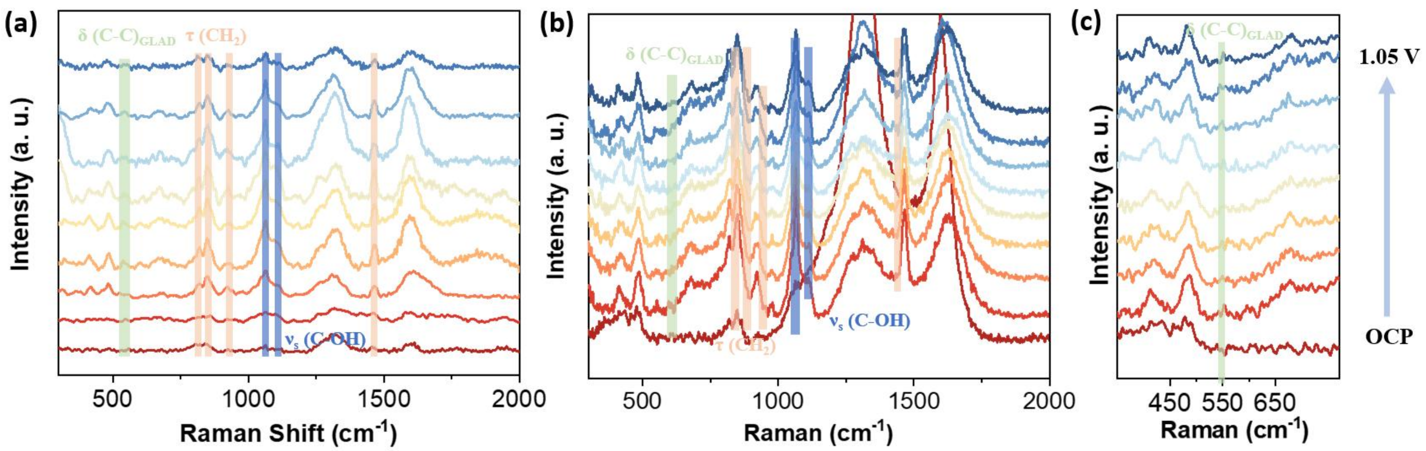

Figure S39. Electrocatalytic *in* situ Raman spectra of PtPbY MNSs detected (a) at catalyst surface and (b) in the electrolyte near the catalyst (different applied potentials). (c) Corresponding zoom-in *in* situ Raman spectra of PtPbY MNSs detected in the electrolyte near the catalyst.

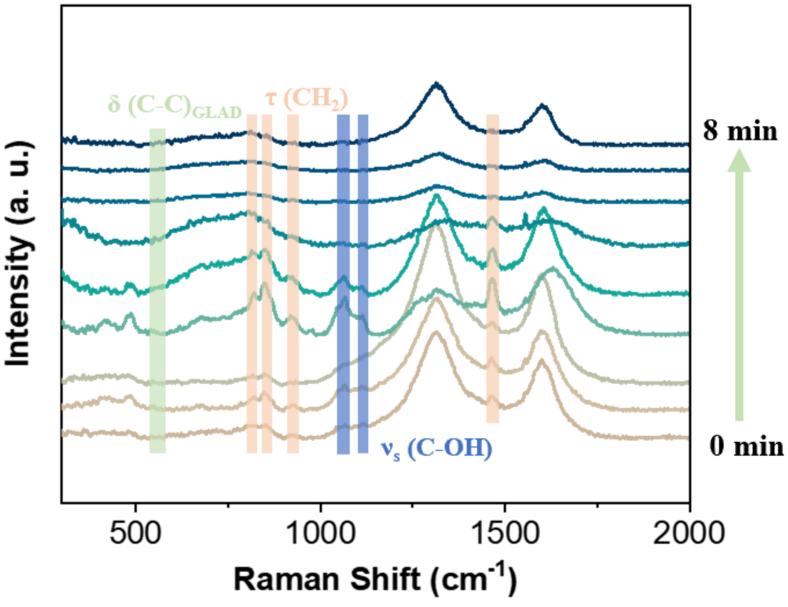

Figure S40. *In situ* Raman spectra of PtPbY MNSs collected in different test times.

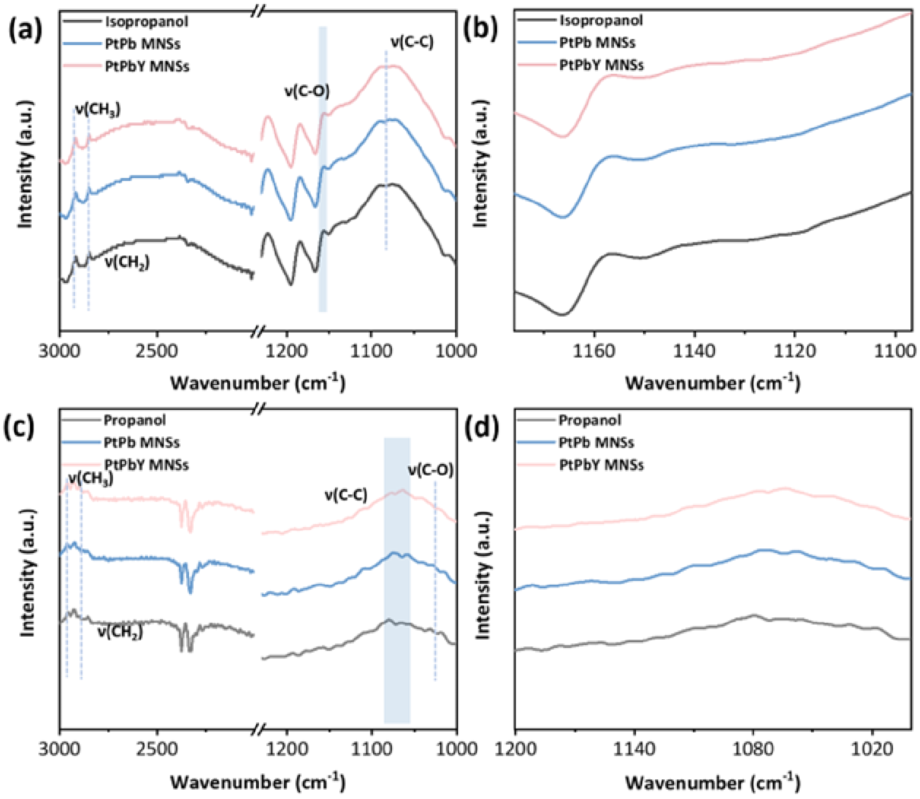

Figure S41. FTIR spectra of (a) isopropanol and (c) propanol adsorbed on PtPb MNSs and PtPbY MNSs. Respective characteristic peak diagrams of (b) isopropanol and (d) propanol.

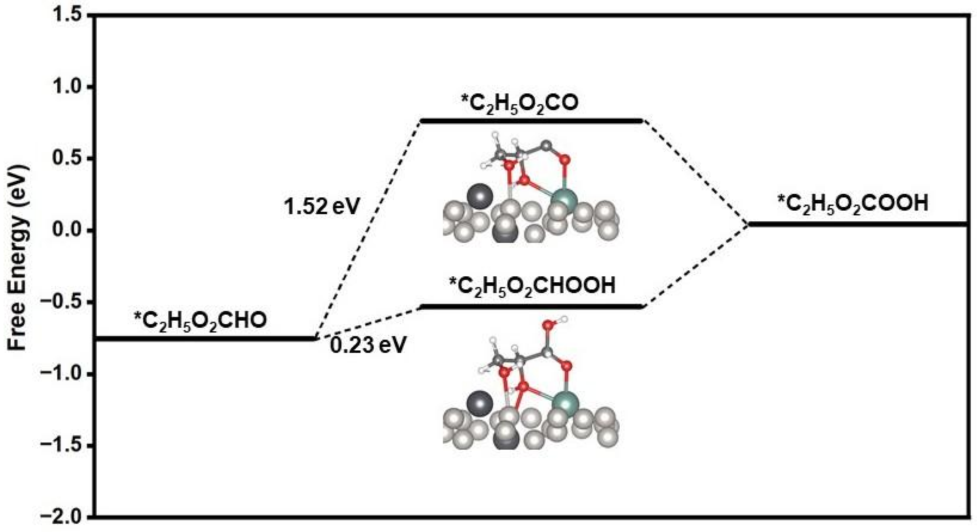

Figure S42. Gibbs free energy of *$C_2H_5O_2CHOOH$ and *$C_2H_5O_2CO$ at U=0 V.

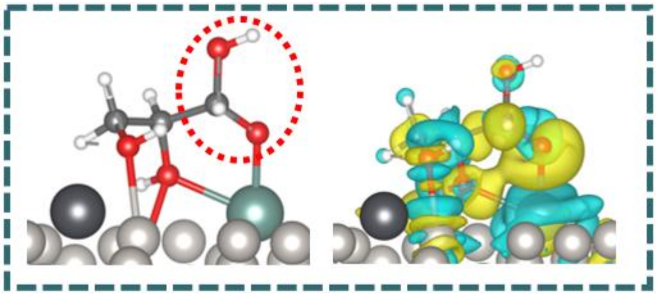

Figure S43. The charge density difference (CDD) analyzed for *$C_2H_5O_2CO$.

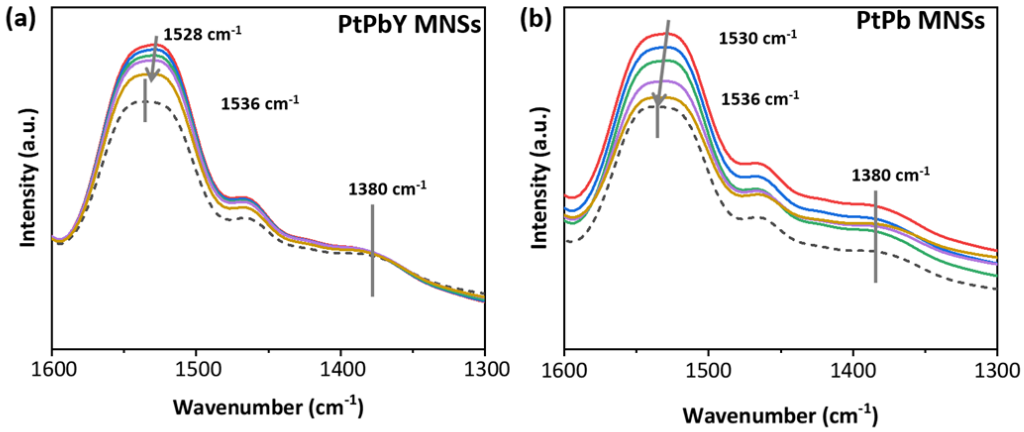

Figure S44. *In situ* FT-IR spectra after the adsorption of GLA for 20 min followed by desorption in different times on (a) PtPbY MNSs and (b) PtPb MNSs. (The dashed lines are the FT-IR spectra of free-state glycerol acid)

Table S1. Comparison of GLA selectivity and yield rate of PtPbY MNSs for GOR electrocatalysis with the electrocatalysts reported in literature.

Catalyst	electrolyte	Selectivity (%)	Production rate (μmol $mg_{cat.}^{-1}$ h^{-1})	Ref.
**PtPbY MNSs**	**1.0 M KOH + 0.5 M GLY**	**72.5**	**656.6**	**This work**
Ni_3Sn	0.5 M KOH + 0.5 M GLY	62.00	352.0	*Small* **2024**, *20*, 2401872
Pd/NF	1.0 M NaOH + 0.1 M GLY	70.50	36.20	*Molecules* **2024**, *29*, 3890
Pt/ZrO_2-350	0.1 M NaOH + 0.1 M GLY	64.90	277.10	*Chem. Eng. J.* 2023, 468, 143623
Pt-TiO_2	0.5 M H_2SO_4 + 0.1 M GLY	29.30	56.01	*Appl. Catal. B Environ. Energy* **2020,** *273*, 119037
WO_3 NSs	0.5 M H_2SO_4 + 0.1 M GLY	73.00	0.0323	*Angew. Chem. Int. Ed.* **2024**, *63*, e202319685
10wt %Pt−C eO$_2$/CNT	1.0 M KOH + 0.1 M GLY	50.00	30.71	*ChemCatChem* **2022**, *14*, e202200509
Pd_{OCTA}	1.0 M KOH + 0.1 M GLY	42.00	435.75	*ACS Appl. Nano Mater.* **2023**, *6*, 11211
O-Pt_3Mn	1.0 M KOH + 0.1 M GLY	67.00	214.40	*ACS Nano* **2025**, *19*, 7154
MoOx/Pt	1.0 M KOH + 0.1 M GLY	73.00	23.60	*Small* **2021**, *17*, 2104288
PtAu/NF	1.0 M KOH + 0.5 M GLY	45.00	310.81	*Energy Environ. Sci.* **2024**,*17*, 4205
